# Hybrid quantum-classical machine learning potential with variational quantum circuits

**DOI:** 10.1039/d6ra07180e

**Published:** 2026-10-26

**Authors:** Soohaeng Yoo Willow, D. ChangMo Yang, Kwang S. Kim, Chang Woo Myung

**Affiliations:** a Department of Energy Science, Sungkyunkwan University 2066 Seobu-ro Suwon Gyeonggi-do 16419 Republic of Korea sy7willow@skku.edu; b Department of Chemistry, Ulsan National Institute of Science and Technology 50 UNIST-gil Ulsan 44919 Republic of Korea kimks@unist.ac.kr; c Department of Energy, Sungkyunkwan University 2066 Seobu-ro Suwon Gyeonggi-do 16419 Republic of Korea cwmyung@skku.edu; d Department of Quantum Information Engineering, Sungkyunkwan University 2066 Seobu-ro Suwon Gyeonggi-do 16419 Republic of Korea

## Abstract

Machine learning potentials (MLPs) based on equivariant message-passing neural networks have achieved state-of-the-art accuracy for atomistic simulation, yet integration of variational quantum circuit (VQC) readouts into such frameworks remains unexplored for condensed-matter systems. Here we employ and characterize a hybrid quantum-classical MLP (HQC-MLP) by coupling an *E*(3)-equivariant message-passing network with VQC readout layers for predicting DFT energies and forces of liquid silicon (64 atoms, periodic cell, 2000–3000 K). We benchmark five VQC Ansatz architectures differing in entangling gate type (controlled-Z (CZ) *vs.* controlled-X (CX)) and circuit structure against a classical baseline. All HQC-MLP variants reproduce PBE-DFT energies with test-set RMSEs of ∼3.5 meV per atom when evaluated using exact expectation values, while entangled circuits exhibit faster training convergence in epoch count, although not in wall-clock time. CZ-based entangling gates achieve lower validation losses than their CX-based counterparts. This trend is consistent with two factors: the phase-preserving nature of CZ gates for amplitude-encoded features and their reduced effective entangling depth, since the final CZ layer commutes with the Pauli-Z measurement operators. Both molecular dynamics and Monte Carlo simulations accurately reproduce the DFT radial distribution function at 2000 K, despite the models being trained exclusively on 3000 K configurations; molecular dynamics also reproduces the Si–Si–Si bond-angle distribution. Finite-shot analysis further shows that achieving an RMSE of approximately 2 meV per atom relative to shot-noise-free expectation values requires about 5 × 10^4^ shots per circuit execution. This corresponds to approximately 2.56 × 10^8^ shots for a single configuration force evaluation, making direct force calculations prohibitively expensive on current noisy intermediate-scale quantum (NISQ) hardware and thereby motivating the proposed Monte Carlo protocol. Finally, the cutoff-radius sensitivity analysis reveals that HQC-MLP exhibits truncation behavior comparable to that of the classical MLP.

## Abstract 2

Equivariant networks with variational quantum circuit readouts yield an accurate hybrid potential for liquid Si, transferable from 3000 to 2000 K.

## Graphical abstract

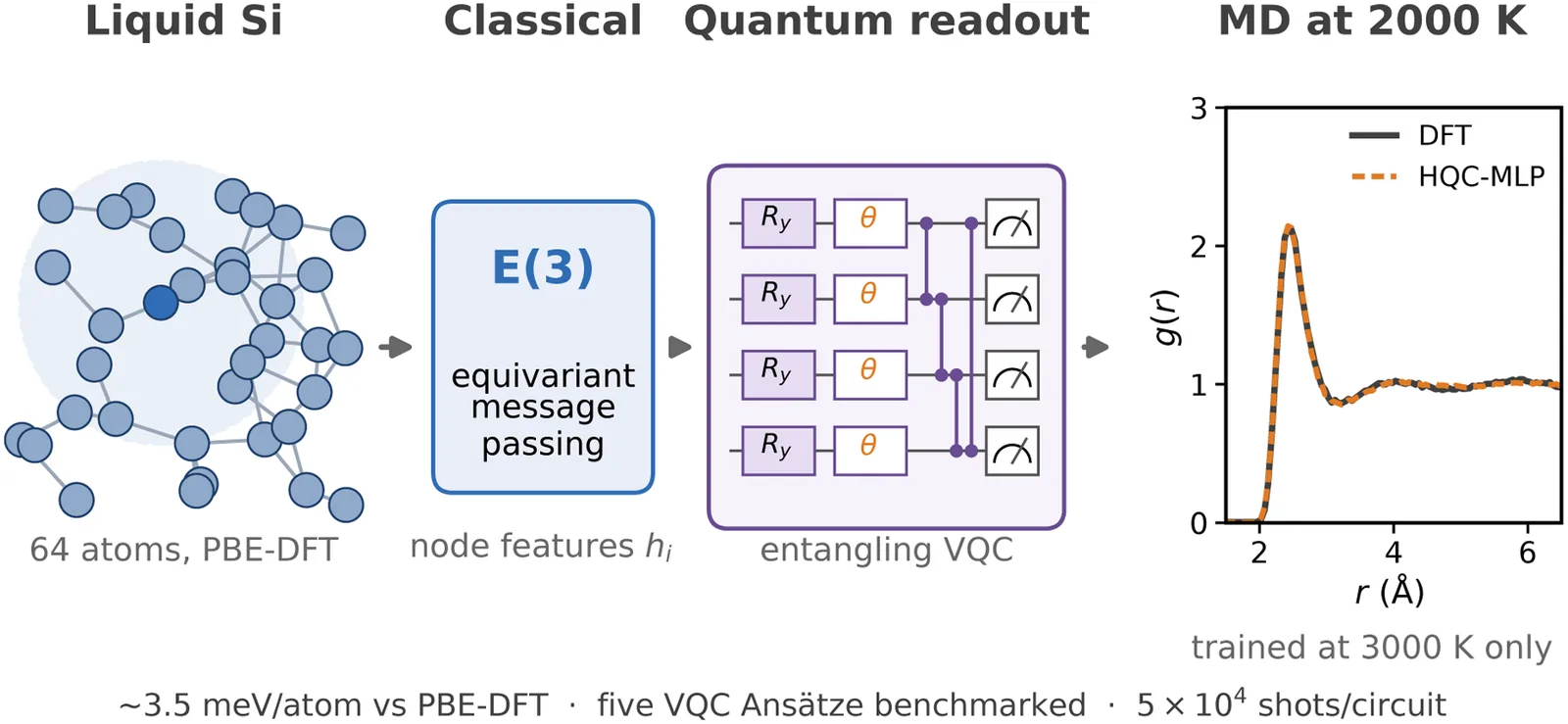

## 1 Introduction

Quantum Machine Learning (QML) has emerged as a significant research frontier, aiming to integrate the principles of quantum computation with the adaptive frameworks of machine learning. The central premise of QML is its potential to leverage quantum phenomena, such as superposition and entanglement, to process information in high-dimensional Hilbert spaces. This capability is theorized to offer substantial advantages over classical models for tasks involving intricate data correlations, leading to active investigation into its application for optimization, classification, sampling techniques, molecular modeling, and combinatorial molecular design.^1–12^

However, despite the growing enthusiasm surrounding QML, there is also skepticism about its practical utility, especially in the near term. Many quantum models have struggled to outperform their classical counterparts in key areas, and the exact role of “quantumness” in improving performance remains an open question.^13^ Studies have shown that while quantum circuits can induce a mapping from classical input features to measured outputs, the inductive bias provided by quantum systems and the problems for which they are particularly well suited are still not fully understood. Classical baseline models outperform prototypical quantum models on small-scale benchmark datasets, and no clear advantage of quantum entanglement in QML has been found for generic learning tasks. As such, QML research remains in a stage of exploration, with both its potential and limitations yet to be fully characterized.

The simulation of materials at the atomic scale presents a domain where hybrid quantum-classical approaches deserve particular scrutiny. Traditional *ab initio* methods face prohibitive scaling: density functional theory scales as 
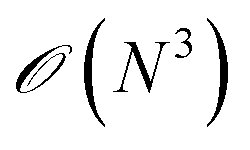
 and coupled-cluster (CC) theory with single, double, and perturbative triple excitations [CCSD(T)] as 
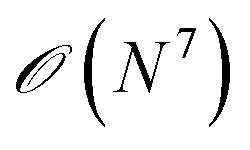
 with system size *N*. Over the past two decades, classical machine learning potentials (MLPs) have emerged as a revolutionary tool,^14–24^ evolving from feed-forward neural networks^25,26^ and Gaussian approximation potentials^27–39^ to graph neural networks.^40–47^ Despite their remarkable success in large-scale materials simulations, classical MLPs still face challenges in capturing non-local electron correlations, and each configuration from *ab initio* molecular dynamics requires significant computational investment. This creates a natural question: can quantum circuits operating natively in Hilbert space provide a more efficient representation of quantum mechanical energy landscapes?

Our goal is not to demonstrate that HQC-MLPs reduce the overall computational cost relative to direct DFT calculations; when finite-shot measurement overhead is included, the inference cost of the hybrid model can exceed that of DFT for the small system sizes studied here. Rather, we employ the HQC-MLP framework as a tool to investigate three well-defined questions: (1) Which VQC architectures integrate most effectively with *E*(3)-equivariant message-passing neural networks? (2) What measurement-shot budgets must near-term quantum devices satisfy for practical deployment? and (3) Does the choice of entangling gate have a systematic, physically interpretable effect on training dynamics and generalization? These questions are answerable with current hardware-simulation tools and provide an empirical and methodological foundation for future VQC-based MLP development.

The potential for quantum advantage in QML is often linked to the ability to recognize hidden patterns in quantum data. Non-local quantum measurements can extract underlying parameters from quantum datasets using only a few samples.^48^ In the context of applying QML to MLPs, referred to as quantum MLPs (QMLPs), the primary goal is to demonstrate quantum advantage by surpassing the performance of classical state-of-the-art MLP models. However, the challenge arises from the fact that the local chemical environment, which is inherently classical, must be mapped onto qubits. This poses a disadvantage, as no known quantum speedup exists for such classical-to-quantum data transformations.^49–52^ Despite this challenge, several studies suggest that polynomial advantage may still be possible for purely classical problems using quantum algorithms.^48,53–56^ However, a clear understanding of how quantum advantage would manifest itself in classical tasks such as QMLP has yet to emerge. For this reason, it is crucial to investigate potential quantum advantages in two key areas: data embedding and the variational Ansatz. When classical local chemical environments are embedded into qubits, the effectiveness of the embedding scheme becomes critical. Furthermore, understanding whether exploiting quantum entanglement can improve machine learning performance during training is another essential aspect. The construction of an effective variational Ansatz circuit with activation functions for potential energy and force regression is crucial for exploring the quantum advantage in QMLPs.^57,58^ Building an equivariant quantum neural network (QNN) is also important for probing the quantum advantage in QMLPs.^13,51,58^ These aspects require careful consideration to elucidate how quantum resources may offer tangible benefits over classical approaches.

Recent studies have demonstrated the potential of quantum machine learning for molecular force field generation. Variational quantum circuits have been employed within hybrid quantum-classical workflows to learn potential energy surfaces for small molecules including H_2_, LiH, Ar_2_, and H_2_O, achieving accuracy competitive with fully classical models.^50,59,60^ We note that in these hybrid schemes the quantum circuit constitutes the entire trainable regression model, while the classical processing is limited to input encoding and parameter optimization. This line of work was extended by training classical machine learning potentials on data computed from quantum processors, with the investigation of noise tolerance and application of the method to individual water molecules and small water clusters containing up to 32 molecules.^61^ More recently, geometric symmetries have been incorporated into QNNs, with the development of invariant architectures for LiH, H_2_O, and water dimers that exhibited improved trainability and generalization.^58^

In the studies above, the trainable model is realized entirely by a quantum circuit, and the systems considered involve at most a handful of internal degrees of freedom.^50,58^ Extending such approaches purely based on quantum circuits to condensed-matter systems is challenging, since the model must describe a far larger number of bonds, bond angles, and torsional interactions while remaining computationally tractable. The HQC-MLP framework adopted here (Fig. 1) addresses this limitation through a different partitioning of the learning task. The VQC operates on the compact invariant node feature vectors produced by the classical *E*(3)-equivariant message-passing network, rather than on the atomic structure directly. This design substantially reduces the quantum computational cost while preserving the structural information required for accurate potential energy predictions.

**Fig. 1:**
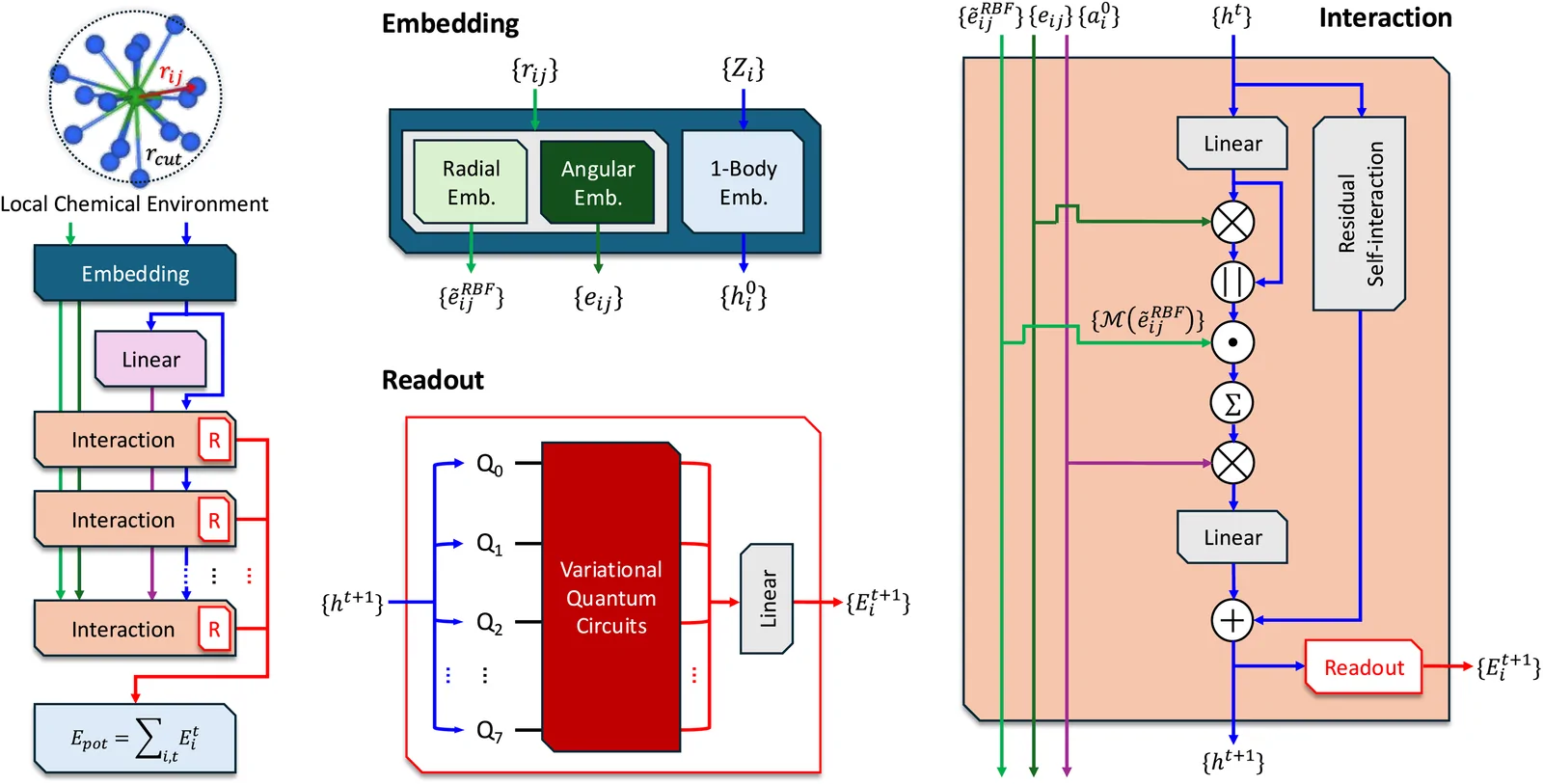
Architecture of the hybrid quantum-classical machine learning potential (HQC-MLP). The HQC-MLP model consists of an embedding layer and interaction layers. Each interaction layer includes a readout layer that predicts output values. In the embedding layer, node features (*h*_*i*_) are initialized based on the atomic numbers of the nodes {*Z*_*i*_}, while edge features *e*_*ij*_ and radial basis vectors *ẽ*^RBF^_*ij*_ are assigned based on pairwise vectors ***r***_*ij*_ = ***r***_*j*_ − ***r***_*i*_. The interaction layer updates node features in a ResNet-like scheme. In the CMLP model, the readout layer applies a non-linear transformation to the node features, generating output values that represent the energy value associated with each node. In the HQC-MLP model, the AngleEmbedding module encodes classical node features into quantum states, while the variational quantum circuits (VQC) process these states, yielding an effective mapping from classical node features to measured outputs.^2,3^ It includes a feature map module, which takes the node features as input, and an Ansatz module, which contains trainable parameters *θ*. Five different VQC Ansatz modules in Fig. 2 were tested.

To the best of our knowledge, this work presents the first application of HQC-MLP methods to a condensed matter system, namely liquid silicon with 64 atoms in a periodic unit cell. This is a substantial increase in system size and complexity relative to the small molecular systems studied previously.^50,59,60^ The target backends for near-term deployment are IonQ Harmony (11 qubits) and IonQ Aria (25 qubits), which guided our circuit design choices. The quantum circuits were evaluated 288 000 times per training epoch (five interaction layers × 64 atoms × 900 training configurations), demonstrating that batched vectorized execution *via* PennyLane^62^ makes training computationally feasible.

Here, we present a systematic architectural study making four concrete contributions. First, we demonstrate that VQC readouts can replace classical readout layers in an *E*(3)-equivariant MPNN without degrading final prediction accuracy, while consistently accelerating training convergence in epoch count, and we show that entanglement—rather than VQC structure alone—is responsible for this effect (demonstrated by the no-entanglement Basic circuit control).

Second, we identify a consistent trend in which CZ-based circuits generalize better than CX-based circuits across the five architectures tested, and provide a mechanistic interpretation based on two consistent factors: the CZ gate's phase-preserving action on amplitude-encoded features,^63,64^ and the reduced effective entangling depth arising from the commutation of the final CZ layer with the Pauli-Z measurements. Third, we quantitatively assess the impact of finite-shot sampling and find that achieving an RMSE of approximately 2 meV per atom relative to shot-noise-free expectation values requires about 5 × 10^4^ shots per circuit execution. Fourth, we provide a systematic cutoff radius sensitivity analysis and a discussion of scalability prospects for larger feature vectors and deeper circuits.

## 2 Hybrid quantum-classical machine learning potential

The objective of our model is to learn a mapping from the positions {***r***_*i*_} and chemical species {*Z*_*i*_} of the *i*-th atom of a system to its total potential energy1
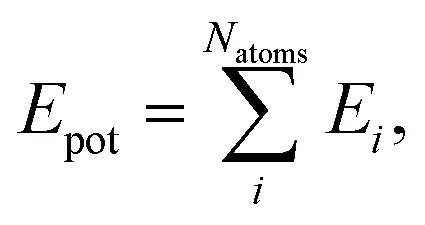
where *E*_*i*_ denotes the atomic energy contribution obtained by aggregating the per-layer readout outputs (Fig. 1). The corresponding atomic forces are computed as ***f***_*i*_ = −∇_*i*_*E*_pot_. The model is energy-conserving and adheres to *E*(3) symmetry: *E*_pot_ is invariant under translation, rotations, and reflections, while forces are equivariant.

### 2.1 *E*(3)-equivariant message-passing framework

The HQC-MLP architecture (Fig. 1) is based on an *E*(3)-equivariant message-passing neural network (MPNN). A function *F* is *E*(3)-equivariant if, for any rotation ***R*** and translation ***t***: *F*(***R***(***x***_*i*_) + ***t***) = ***R***(*F*(***x***_*i*_)) for vector outputs (such as forces), or *F*(***R***(***x***_*i*_) + ***t***) = *F*(***x***_*i*_) for scalar outputs such as energy.^40–43^ In this framework, each atomic structure is converted into a graph where atoms are nodes and the edges connect neighbors within a cutoff radius *r*_cut_. MPNN iteratively updates the node features through message-passing operations employing steerable filters

2 *S*_*m*_^(*l*)^(***r***_*ij*_) = *R*^(*l*)^(*r*_*ij*_)*Y*_*m*_^(*l*)^(***r̂***_*ij*_),

where *Y*_*m*_^(*l*)^(***r̂***_*ij*_) are real spherical harmonics and *R*(*r*_*ij*_) are learnable radial functions. This construction makes the convolution operations equivariant under *O*(3) (rotations and reflections).

Node features ***h***_*i*_^(*t*)^ are updated in a ResNet scheme:^65^

3 ***h***^(*t*+1)^ = *f*_SI_(***h***^(*t*)^) + *f*_conv_(***h***^(*t*)^, ***e***_*ij*_, ***ẽ***^RBF^_*ij*_),

where *f*_SI_ is an element-specific self-interaction and *f*_conv_ is a continuous-filter convolution.^66^ Radial basis vectors *ẽ*^RBF^_*ij*_ are computed using 8 Bessel basis functions with polynomial envelope (*p* = 2). The algorithm flowchart of the hybrid model is provided in SI Fig. S3.

### 2.2 Variational quantum circuit readout

In standard *E*(3)-equivariant MPNNs, the readout layer maps invariant node features (rotational order *l* = 0) to a scalar atomic energy *via* a classical SiLU-gated dense layer. In our HQC-MLP model, we replace this classical readout part with a VQC. The key motivation is that the invariant node features encode local chemical environment information, and the quantum operations acting on these features introduce entanglement between qubits. This entanglement is a form of correlation generated by the circuit itself and couples the encoded features through products of their encoded angles, on a sparse pattern fixed by the gate layout. The classical readout also couples features, through the dense, learned linear layers around its element-wise SiLU activation; the two readouts differ in the form of the coupling (Section 3.2). We emphasize that the term ‘quantumness’ here refers specifically to the entanglement generated between qubits during computation, and not to any inherent quantum property of the input features.

The VQC module consists of three stages: (1) *Feature encoding*: invariant node features 
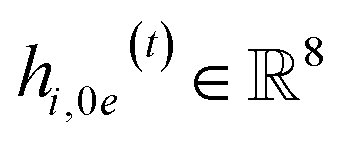
, an 8-dimensional vector per atom, are encoded onto *N* = 8 qubits using AngleEmbedding, where each feature element parameterizes a single-qubit RY rotation gate. (2) *Variational Ansatz*: a parameterized Ansatz applies a trainable linear transformation through quantum operations in Hilbert space. The trainable parameters *θ* are optimized concurrently with the classical components. (3) *Measurement*: entanglement-modified node features are extracted by measuring the Pauli-Z expectation value on each qubit: 〈*Z*_*k*_〉 for *k* = 0, …, 7.

To systematically study the impact of circuit structure, we designed and tested five distinct Ansatz architectures (Fig. 2), differing in two primary aspects, the choice of entangling gate (CX*vs.*CZ) and the inclusion or exclusion of a quantum Fourier transform (QFT) block. Specifically, (1) the Basic circuit (no entangling gates, no QFT) is a baseline using only single-qubit rotations, providing a circuit with no entanglement; (2) the CX circuits (BasicEntanglerCX and QFTEntanglerCX) employ controlled-X entangling gates; (3) the CZ circuits (BasicEntanglerCZ and QFTEntanglerCZ) employ controlled-Z entangling gates. The CZ and CX gates are unparameterized, so all five circuits carry identical numbers of trainable parameters and differ only in the presence and type of the entangling operation. We note that the final CZ ring commutes with the terminal Pauli-Z measurements and is retained for structural symmetry with the CX circuits; its consequences for the effective circuit depth are discussed in Section 4.1.

**Fig. 2:**
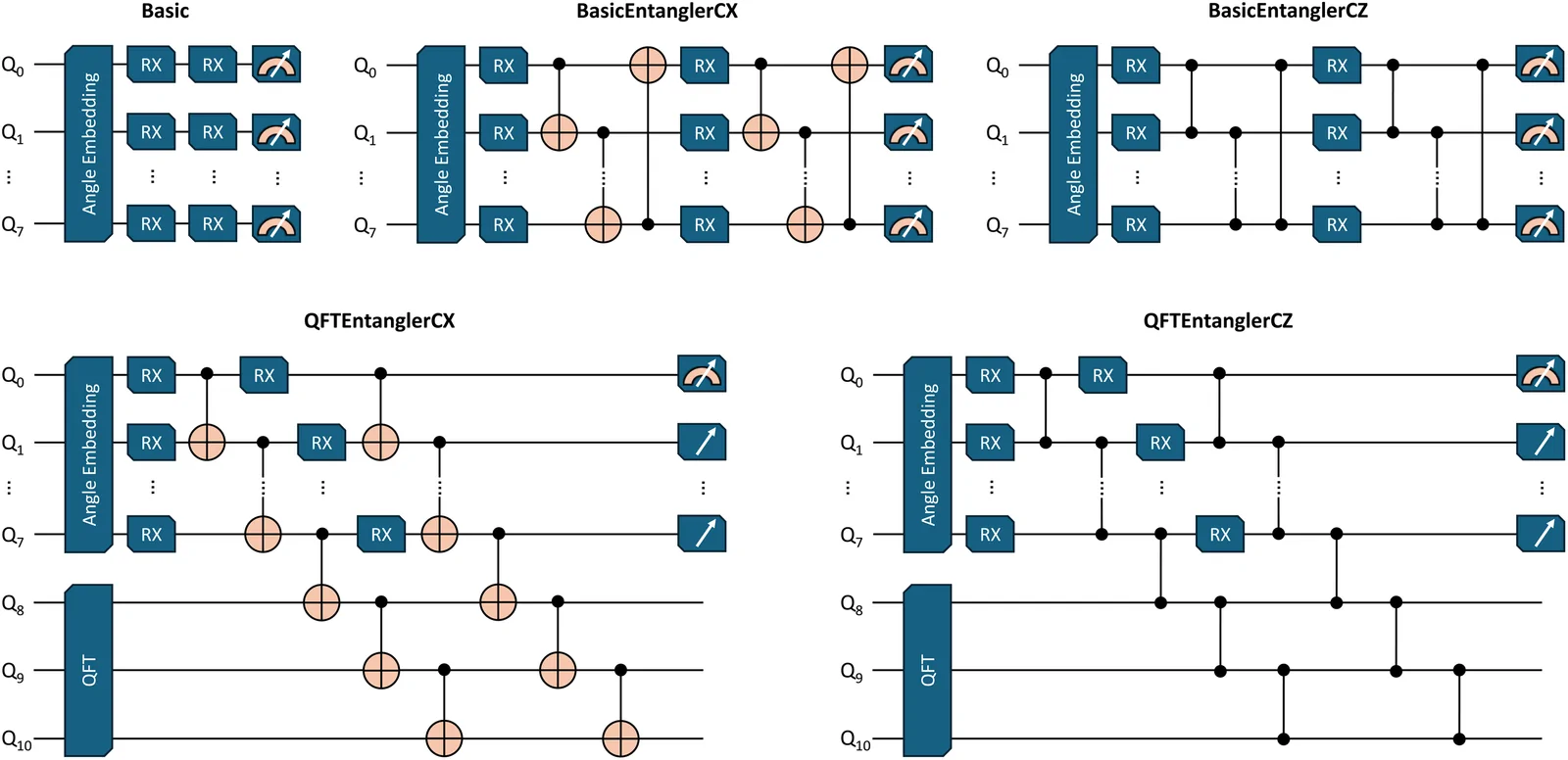
Diagrams of five distinct Ansatz modules, shown with 8 qubits for AngleEmbedding, 3 qubits for QFT, and, for illustration, 2 layers of trainable rotational RX gates (3 layers were used in training). The AngleEmbedding module serves as the feature map across all quantum circuits, each of which uses one of five Ansatz module variations. The Ansatz modules are categorized by the type of the entanglement and by whether quantum Fourier transform (QFT) is incorporated. Entanglement between qubits is achieved using either the controlled-NOT (CX) or the controlled-Z (CZ).

The QFT variants follow West *et al.*,^13^ who construct rotationally equivariant models for two-dimensional image data upon the QFT and report both absence of barren plateaus and a marked empirical advantage over generic circuits. In their construction the QFT establishes the equivariance itself, whereas here it is tested only as a candidate inductive bias, since the *E*(3) symmetry of the present model is enforced entirely by the classical message-passing network. The QFT acts on a 3-qubit ancillary register distinct from the 8 encoding qubits, with the entangling chain extending across both registers. Measurement is performed only on the encoding qubits and the ancilla is traced out, so the QFT never transforms the AngleEmbedding state directly. It instead prepares an auxiliary subsystem in a Fourier basis whose entanglement with the encoding register modifies the reduced state from which the atomic energy is read out. Since the ancilla is initialized in |0〉^⊗3^, this preparation is operationally equivalent to a uniform superposition (QFT|0〉^⊗3^ = |+〉^⊗3^), so the QFT variants probe the effect of ancilla coupling through the extended entangling chain rather than the Fourier phase structure itself. This arrangement preserves the encoded amplitude structure and keeps the total at 11 qubits, within the capacity of IonQ Harmony. We note that the QFT variants accordingly differ from their counterparts both in the QFT block and in the length of the entangling chain, although the trainable parameter count is unchanged.

Function class of the readout. Because the features are encoded once, by RY rotations, each output 〈*Z*_*k*_〉 is a linear combination of products of 1, cos *x*_*i*_, and sin *x*_*i*_ over the input features *x*_*i*_;^67^ the entangling gates determine which products appear. In the Basic circuit, the three RX rotations on each qubit combine into one, giving 〈*Z*_*k*_〉 = cos(*θ*_1*k*_ + *θ*_2*k*_ + *θ*_3*k*_)cos *x*_*k*_, so the Basic readout is exactly a cosine activation. BasicEntanglerCZ and BasicEntanglerCX share the same layer structure and differ only in the entangling gate, yet couple the features differently. CZ gates are diagonal, so they commute with one another and with the *Z* measurement: each layer couples a qubit only to its nearest neighbors, the final layer has no effect, and each output couples five of the eight features. The sequential CNOT ring instead passes information around the whole ring within one layer, so every output couples all eight. All five circuits carry the same 24 trainable parameters per interaction layer. The circuits add 120 trainable parameters to the model (24 parameters per layer across five layers).

Each Ansatz was constructed with three repeated layers and implemented using PennyLane^62^ with a total of 8 qubits for the main circuit and an additional 3-qubit subsystem for the QFT block when present. All quantum circuits were classically simulated during training; our target near-term hardware backends are IonQ Harmony (11 qubits) and IonQ Aria (25 qubits), whose qubit counts constrained the circuit design.

## 3 Methods

### 3.1 Generation of training and validation datasets

The dataset was generated from a single Born–Oppenheimer molecular dynamics (BOMD) simulation of liquid silicon at 3000 K using DFT. The system consisted of 64 silicon atoms in a periodic cubic cell (10.862 Å × 10.862 Å × 10.862 Å). DFT calculations used the hybrid Gaussian and plane-wave (GPW) method,^68^ GTH norm-conserving pseudopotentials,^69,70^ PBE exchange–correlation functional,^71^ DZVP basis set, 400 Ry charge density cutoff, and electronic convergence *ε*_SCF_ ≤ 5 × 10^−5^, all within QuickStep/CP2K.^72,73^ BOMD was run with velocity Verlet integration^74^ at 0.5 fs timestep and a Nosé–Hoover-chain thermostat.^75^ After 10 ps equilibration, 18.8 ps of data collection yielded 1880 configurations sampled every 10 fs. These configurations were randomly shuffled and divided into training (900), validation (100), and testing (880) sets, reducing configurational redundancy and avoiding data leakage.

An additional BOMD simulation was performed at *T* = 2000 K following the same protocol used for the *T* = 3000 K simulation. This trajectory served exclusively as a structural reference for calculating the radial distribution functions presented in Fig. 4 and S8 and the angular distribution function in Fig. S10, and was not used for the training, validation, or testing. The 3000 K comparisons (Fig. S9 and S11) use the 3000 K DFT trajectory described above.

Hyperparameters were selected through a systematic optimization carried out using the classical neural network model. The architectural choices for variational quantum circuits (number of qubits, circuit depth, gate types) were design decisions informed by hardware constraints (IonQ Harmony: 11 qubits; IonQ Aria: 25 qubits). The validation set was used to optimize hyperparameters, monitor convergence, and select the early-stopping checkpoint at the minimum of the validation loss. The test set (880 configurations) was never used during training or model selection, and all reported metrics were evaluated on it at the selected checkpoint.

### 3.2 Model architecture and training

The model architecture includes 5 interaction layers. The hidden feature dimension in each layer is represented in e3nn notation^40^ as “64x0e + 64x0o + 64x1o + 64x1e + 8x2o + 8x2e”. Radial basis vectors *ẽ*^RBF^_*ij*_ use 8 Bessel basis functions with polynomial envelope (*p* = 2),^66^ input into a multilayer perceptron 
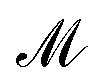
 of size [64, 64], with SiLU non-linearities. In the CMLP readout, the 64 invariant channels of each interaction layer are projected onto 32 inputs of the SiLU-gated layer before the linear map to the atomic energy; in the HQC-MLP they are projected onto the eight circuit inputs. This gives 2081 trainable parameters per interaction layer for the CMLP readout and 545 for the HQC-MLP readout (Fig. S12). The CMLP readout thus has four times the hidden width and 3.8 times the trainable parameters, and couples the features densely through learned linear maps, whereas the circuit couples them sparsely, on a pattern fixed by the gate layout (Section 2.2). The baseline cutoff was *r*_cut_ = 4.5 Å (∼25 neighbors per atom); additional models for the sensitivity analysis were trained at *r*_cut_ ∈ {3.5, 4.0, 5.0} Å with all other settings fixed. The dimensionless loss function is:4
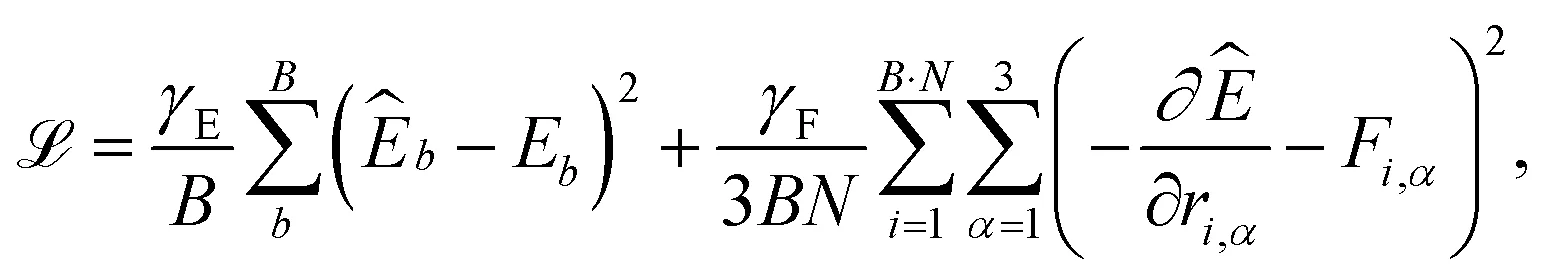
where *B* denotes the batch size and *N* the number of atoms per configuration. *E*_*b*_ and *Ê*_*b*_ are the true and predicted total energies per configuration, respectively, *F*_*i*,*α*_ the force on the atom *i* in the direction *α* ∈ {*x*, *y*, *z*}. The energy loss weighting factor *γ*_E_ was chosen as 1 eV^−2^ and the force loss weighting factor *γ*_F_ was set to 100 (eV Å)^−2^ to ensure the combined loss is dimensionless.

Optimization used AMSGrad,^76^ a variant of Adam,^77^ with *β*_1_ = 0.9, *β*_2_ = 0.999, and *ε* = 10^−8^, a learning rate of 0.01 and a batch size of 20. On-plateau learning rate scheduler (patience 250 epochs, a decay factor 0.9) and exponential moving average (weight 0.99) were used. Float32 precision; NVIDIA GeForce RTX 4090 GPU; 8.6 s per epoch for the CMLP and 10.7–23.2 s per epoch for the HQC-MLPs (Table S1). Training required 288 000 circuit evaluations per epoch (5 layers × 64 atoms × 900 configurations). Software versions: pennylane 0.42.3, jax 0.6.2, jaxlib 0.6.2, matscipy 1.1.1, ase 3.23.0, e3nn-jax 0.20.8. All code and data are publicly available at https://github.com/myung-group/BAM-QML.

### 3.3 Validation of models

#### 3.3.1 Molecular dynamics simulations

NVT MD simulations at *T* = 2000 K using the ASE package^78^ with a time step of 0.5 fs and Nosé–Hoover and Parrinello–Rahman dynamics.^79–81^

#### 3.3.2 Monte Carlo simulations

Canonical MC at *T* = 2000 K using the Metropolis–Hastings algorithm,^82^ requiring only energy evaluation (no forces), to access structural properties under shot-noise conditions.

#### 3.3.3 Cutoff sensitivity

Both HQC-MLP (BasicEntanglerCZ) and CMLP were retrained from scratch at each *r*_cut_ with dataset partitions and random seeds held fixed and a common reduced training budget (see Table 1 caption). Test-set RMSE_E_ and RMSE_F_ are reported.

**Table 1:** Cutoff-radius sensitivity. Test-set energy RMSE_E_ (meV per atom) and RMSE_F_ (eV Å^−1^) for HQC-MLP (BasicEntanglerCZ) and CMLP. All other hyperparameters, dataset partitions, and random seeds held fixed. The cutoff series was trained under a reduced common budget (batch size 5, 8000 epochs; Fig. S6) rather than the production settings of Section 3.2, so absolute RMSEs exceed the converged values reported in Section 4.1 while remaining directly comparable across *r*_cut_ and between architectures

*r*_cut_	HQC-MLP		CMLP	
	RMSE_E_	RMSE_F_	RMSE_E_	RMSE_F_
3.5	9.1	0.27	9.5	0.27
4.0	6.0	0.25	6.2	0.25
4.5	3.7	0.24	3.2	0.23
5.0	2.8	0.22	2.5	0.22

## 4 Results and discussion

### 4.1 Training of HQC-MLP models

To evaluate the models, we generated a dataset of 1880 liquid silicon configurations at 3000 K from a single BOMD trajectory using DFT. Configurations were sampled every 10 fs, then randomly shuffled and split into training (900), validation (100), and test (880) sets. This procedure, drawing from a single long trajectory rather than multiple independent runs, avoids correlation between sets that can arise from shared initial conditions, following standard practices for molecular machine learning systems. Fig. 3 compares the training and validation loss curves for the CMLP and HQC-MLP models. The loss function, described in Section 3.2, uses dimensionless MSE combining energy and force MSE with weighting factors *γ*_E_ = 1 eV^−2^ and *γ*_F_ = 100 (eV Å)^−2^.

**Fig. 3:**
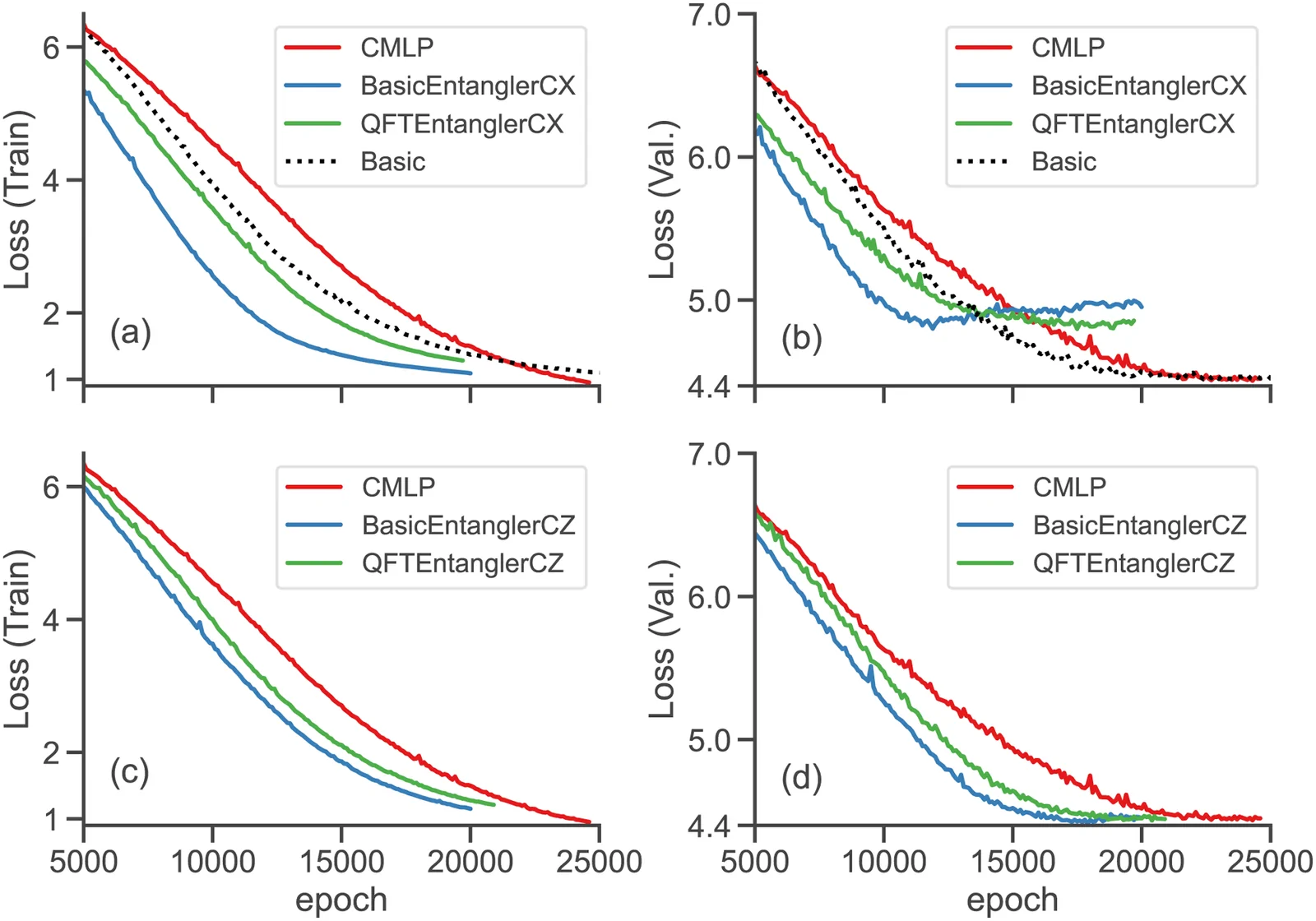
Loss profiles for hybrid quantum-classical machine learning potential (HQC-MLP) models in comparison to a classical machine learning potential (CMLP) model. Loss values are evaluated for (a and c) training and (b and d) validation datasets. The loss function used during training is described in Section 3.2. In the training dataset, loss values continue to decrease. In the validation dataset, however, loss values reach minimum points, indicating the onset of model overfitting to the training data.

A primary observation is that the HQC-MLP models consistently converge faster on the training data than the purely classical model (CMLP) as shown in Fig. 3 and S4. This acceleration is particularly pronounced for CX-based circuits, but it is accompanied by an earlier onset of overfitting, as indicated by a premature increase in their validation loss. In contrast, the Basic HQC-MLP model (no entanglement) follows a training loss trend close to that of the CMLP, indicating that replacing the classical SiLU readout with a variational circuit is not by itself sufficient to accelerate convergence. Because the entangling gates are unparameterized, this comparison holds the number of trainable parameters fixed. The CZ and CX architectures provide a complementary control, since they share connectivity, qubit-to-feature assignment, depth, and trainable parameter count and differ only in the entangling gate, yet exhibit systematically different convergence and generalization behavior. One structural asymmetry should, however, be emphasized. Because the CZ gate is diagonal in the computational basis, it commutes with Pauli-Z measurements. Consequently, the final CZ entangler ring has no effect on the measured expectation values (〈*Z*_*k*_〉). Since this invariance holds for all parameter values, it also leaves all training gradients unchanged. As a result, the CZ circuits effectively contain one fewer measurement-relevant entangling layer than the corresponding CX circuits (two instead of three). The comparison between CZ and CX gate types is therefore partially confounded by a difference in effective entangling depth, rather than reflecting the gate choice alone. Taken together, these comparisons show that the coupling pattern set by the entangling gates, not the parameter count, determines both the convergence rate and the generalization. Whether a classically simulated circuit of this size exhibits inherently quantum behavior is an open question that these controls do not address; the question they answer is which entangling structure provides a useful inductive bias for smooth regression at a fixed parameter count. The comparison with the classical readout sharpens this point. The CMLP readout has four times the hidden width and 3.8 times the trainable parameters of the circuit readout, and its coupling is dense and fully learned, whereas that of the circuit is sparse and fixed by the gate layout (Section 3.2). Yet no circuit needs more epochs than the CMLP. To reach the common validation force error of Table S1, it requires about 14 600 epochs, compared with 9800 for BasicEntanglerCX, 12 800 for Basic and BasicEntanglerCZ, and 12 300 and 12 500 for the two QFT variants. The difference between the readouts therefore lies in the form of the coupling.

We note that this epoch-count speedup does not constitute a wall-clock or computational cost advantage. Each hybrid epoch requires 288 000 circuit evaluations (=1 circuit/layer × 5 layers/atom × 64 atoms/configuration × 900 configurations). We measured the training cost from the time stamps of the training logs, using runs executed consecutively on the same NVIDIA GeForce RTX 4090 GPU with identical training settings (Table S1). An epoch of the eight-qubit HQC-MLPs takes 1.25–1.31 times as long as a CMLP epoch, and an epoch of the QFT variants, whose 11-qubit state vector is eight times larger, 2.35–2.71 times as long. Measured to a common validation force error (RMSE_F_ ≤ 0.222 eV Å^−1^), the smaller number of epochs largely offsets the higher per-epoch cost of the eight-qubit variants, which reach the target in 0.83–1.15 times the CMLP wall time, but not that of the QFT variants, which need about twice as long. The training cost of the eight-qubit variants is comparable to that of the CMLP.

Across the three random seeds tested for each gate type (Fig. S4), the CZ-based models show a consistent trend toward lower minimum validation loss compared to the CX-based models; however, seed-to-seed variability partially overlaps between groups, and a definitive statistical separation would require a larger seed ensemble. The CMLP reaches a final validation loss comparable to the best CZ seed. The CX-based models consistently exhibited earlier and more severe overfitting than the CZ variants, with higher minimum validation loss across all three seeds tested. Comparing the QFT and non-QFT entanglers within each gate family, the QFT block did not produce a systematic generalization advantage. It yields only a modest reduction in minimum validation loss for the CX-based circuits (Fig. 3b) and is essentially neutral for the CZ-based circuits (Fig. 3d). The dominant architectural factor is therefore the choice of entangling gate rather than the presence of the ancillary register. Fig. S5 tracks the energy and force RMSEs on the validation sets as a function of training epoch; these curves are the monitoring signal used to identify the early-stopping checkpoint. Evaluated at that checkpoint on the held-out test set, the model reaches RMSE_E_ ≈ 3.5 meV per atom and RMSE_F_ ≈ 0.22–0.27 eV Å^−1^ relative to the PBE-DFT reference. These values are comparable to, and in some cases better than, early-generation neural network potentials.^25^ A direct architecture comparison under the matched training protocol of Table 1 (*r*_cut_ = 4.5 Å) yields RMSE_E_ = 3.7 meV per atom for HQC-MLP and 3.2 meV per atom for CMLP, a difference near the seed-variability noise floor; both architectures thus demonstrate similar levels of accuracy. At the production settings, the CMLP validation loss converges to a level comparable to the best CZ seed (Fig. 3d), consistent with this conclusion.

To understand the CZ advantage mechanistically, we note that the two gates act qualitatively differently on quantum states. The CZ gate applies a conditional phase flip: it maps |11〉 → −|11〉 while leaving other basis states unchanged, thereby modifying the phase structure of the wavefunction without permuting state amplitudes. This preserves the amplitude information encoded during AngleEmbedding while introducing inter-qubit correlations. In contrast, the CX (CNOT) gate performs a conditional bit flip, actively permuting amplitudes and potentially disrupting the smooth encoding of input features. For the smooth scalar regression task of potential energy surface fitting, amplitude-preserving phase operations provide a more appropriate inductive bias. The reduced effective entangling depth discussed above likely contributes in the same direction. A circuit with fewer active entangling layers spans a smaller function class, and the present experiments cannot disentangle the respective contributions of amplitude-preserving phase action and reduced effective expressivity. Nevertheless, both effects reflect the same underlying principle: circuit expressibility should be matched to the complexity of the learning task.^63,64^ Excessively expressive circuits are more prone to overfitting, whereas circuits with an appropriate degree of entanglement tend to exhibit better generalization.^83^ Isolating these two mechanisms would require a depth-matched control, such as a CZ Ansatz followed by a final rotation layer, which we leave for future work.

### 4.2 Sensitivity to cutoff radius

One motivation for using quantum readouts is to capture non-local atomic interactions beyond the classical cutoff. We therefore explicitly examine whether HQC-MLP exhibits different sensitivity to the cutoff radius *r*_cut_ compared to CMLP.

The baseline value *r*_cut_ = 4.5 Å was chosen to encompass the first two maxima of the DFT radial distribution function of liquid silicon, namely, the first peak (∼2.35 Å) and the broad second maximum lying within the second shell (3.5–5.0 Å), so that the cutoff sphere contains the full first coordination shell and the dominant part of the second. This choice yields ∼25 neighbors per atom on average, balancing environment completeness against computational cost. To assess sensitivity to the cutoff radius, we retrained both HQC-MLP (using BasicEntanglerCZ Ansatz module, the best-performing variant) and CMLP with *r*_cut_ ∈ {3.5, 4.0, 4.5, 5.0} Å. The dataset partitions, random seeds, and a common reduced training budget were held fixed across all models (see the caption of Table 1 for details). Results are summarized in Table 1 and Fig. S6.

Both architectures display the same monotonic trend: energy and force errors decrease smoothly as *r*_cut_ is enlarged, with the steepest gains occurring between 3.5 and 4.5 Å, where the second coordination shell becomes accessible. Beyond 4.5 Å, the improvement persists but is much smaller (∼1 meV per atom at *r*_cut_ = 5.0 Å, *versus* ∼5 meV per atom between 3.5 and 4.5 Å), consistent with the locality of bonding in liquid silicon. The training curves in Fig. S6 show that both models also converge at comparable rates at every tested cutoff, indicating that the cutoff-driven error is dominated by the information content of the local chemical environment.

Since the differences between the two models (≲ 0.5 meV per atom in RMSE_E_) lie near the noise floor expected from random-seed variability, we conclude that the VQC readout does not confer additional resilience to incomplete local chemical environment information for this weakly correlated, predominantly covalent system. This null result is consistent with the physical character of liquid silicon, whose bonding is dominated by short-range, near-tetrahedral correlations that are already well captured within the message-passing radius. Whether this picture changes for systems in which non-local electron correlations are physically essential, such as strongly correlated oxides, Mott insulators, or systems near a metal–insulator transition, remains an open question and an important direction for future work.

### 4.3 Molecular dynamics simulations

To validate the trained HQC-MLP, we performed 100 ps (NVT) molecular dynamics (MD) simulations at *T* = 2000 K using the ASE package.^78^ Simulating liquid silicon at high temperatures is a well-known challenge, as standard empirical potentials such as the Stillinger–Weber,^84^ Lenosky,^85^ Bazant,^86^ and Tersoff^87^ models are known to inadequately describe its structural properties; DFT provides an accurate reference. A key test for any new potential is total energy conservation. All HQC-MLP and CMLP models conserved energy throughout the full trajectory (see Fig. S7), confirming a smooth learned potential energy surface. In a simpler version of the HQC-MLP architecture (SI Section S2), the VQC readout failed to preserve the short-range repulsive interactions between Si atoms, producing an unphysical peak at *r* < 2 Å in the radial distribution function (RDF) (Fig. S2). Notably, this failure was invisible to the static energy and force RMSE, surfacing only in the dynamical simulation. It motivated the improved architecture shown in Fig. 1.

Fig. 4 presents the RDF for liquid silicon at *T* = 2000 K, comparing the HQC-MLP (BasicEntanglerCX) model with DFT BOMD. This comparison assesses the model's transferability to a lower temperature, outside the training distribution, as it was trained exclusively on DFT configurations sampled at 3000 K, with no 2000 K configurations included in the training set. The DFT-based BOMD simulations were performed for 10 ps equilibration and 18 ps data production. The HQC-MLP simulations were run for 100 ps. We show results for HQC-MLP(BasicEntanglerCX) here as a conservative baseline comparison. The remaining entangled variants – BasicEntanglerCZ, QFTEntanglerCZ, and QFTEntanglerCX – are shown in Fig. S8 and are in equally good agreement with the 2000 K DFT reference; the principal peak at *r* ≈ 2.35 Å and a broad second shell between 3.5 and 5.0 Å are faithfully reproduced in all cases. RDF accuracy therefore does not discriminate among the four entangled architectures: the choice of entangling gate affects training dynamics but not equilibrium structural properties of liquid silicon at this state point. Notably, all models were trained exclusively on configurations sampled at 3000 K. Therefore, the agreement with the independent DFT reference at 2000 K provides a direct assessment of the model's transferability to a thermodynamic state point outside the training distribution.

**Fig. 4:**
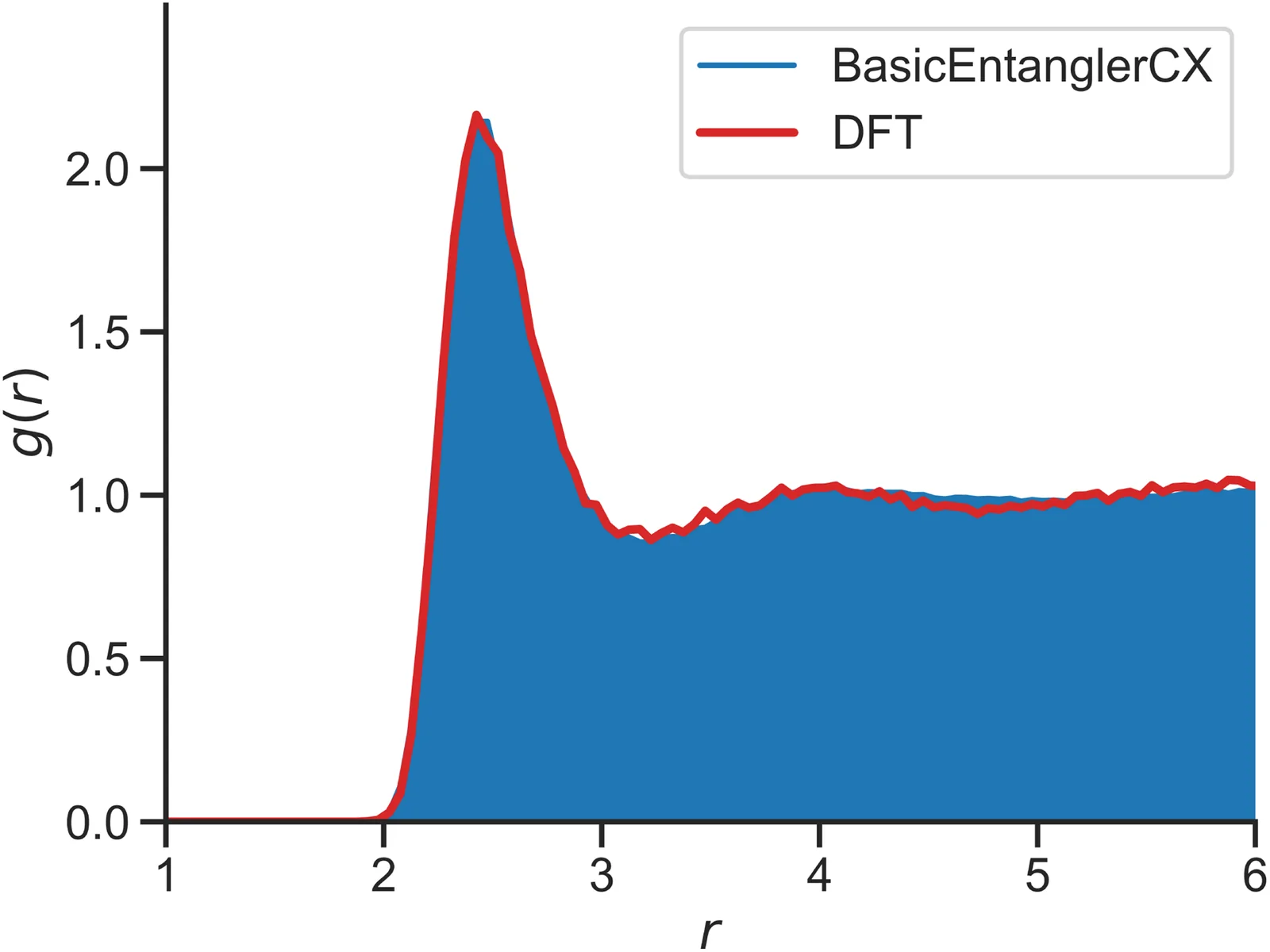
Radial distribution function, *g*(*r*), for liquid silicon at *T* = 2000 K: transferability test for models trained exclusively on 3000 K configurations. The DFT reference was obtained at 2000 K and was not used for training or model selection. The plot compares the result from the HQC-MLP(BasicEntanglerCX) model (blue filled area) with DFT BOMD reference (red line). Both curves were obtained from NVT-MD simulations of a 64-atom system in a cubic cell (*a* = 10.862 Å). The HQC-MLP curve is from a 100 ps NVT-MD simulation using the ASE Nosé–Hoover thermostat; the DFT reference is from a BOMD simulation (18 ps production after 10 ps equilibration at *T* = 2000 K). RDFs for the remaining entangled variants at 2000 K are shown in Fig. S8; the non-entangled Basic circuit, evaluated at the training temperature (3000 K) as an in-distribution check, is shown in Fig. S9.

The non-entangled Basic circuit was evaluated separately at *T* = 3000 K, the temperature of the training data, and likewise reproduces the DFT RDF (Fig. S9); that comparison serves as an in-distribution consistency check rather than as a test of transferability.

Because agreement in *g*(*r*) does not establish the accuracy of angular correlations, we also compared the Si–Si–Si bond-angle distributions *P*(*θ*) with a common neighbor cutoff of 3.24 Å (Section S9). At 3000 K, the Basic model reproduces the DFT distribution (Fig. S11). At 2000 K, all four entangled variants reproduce both maxima, near 55° and 95°, and the mean coordination number (6.48–6.50, *versus* 6.48 for DFT) (Fig. S10).

### 4.4 Effect of finite measurement shots

The preceding analyses used mathematically exact expectation values from the state vector simulation. To assess the performance on realistic quantum hardware, we examined statistical noise from a finite number of measurement shots, *S*. Fig. 5 quantifies the convergence of the predicted potential energies as a function of 
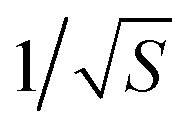
. As expected from sampling theory, RMSE_E_ between the shot-based predictions and the exact expectation values follows 
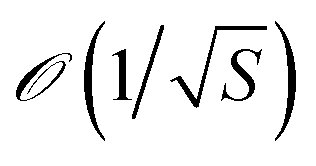
. Using the testing dataset, we found that approximately 5 × 10^4^ shots per circuit execution are sufficient to reduce the sampling-induced error to an RMSE_E_ of about 2.0 meV per atom. This RMSE quantifies the deviation of shot-based predictions from the exact expectation values of the same trained model and therefore represents a source of error that is distinct from the intrinsic model error of RMSE_E_ ≈ 3.5 meV per atom relative to PBE-DFT reported in Section 4.1. Here, a ‘per configuration energy evaluation’ refers to a complete inference pass over all 5 interaction layers and 64 atomic readout circuits for one atomic configuration, corresponding to 320 individual circuit executions. Consequently, at a budget of 5 × 10^4^ shots per circuit, this requires approximately 1.6 × 10^7^ total shots for a single configuration energy evaluation. We further emphasize that sampling-induced RMSE of 2.0 meV per atom should not be interpreted as ‘chemical accuracy’ in the strict sense. Conventionally, chemical accuracy refers to agreement with an exact reference, such as experimental measurement or FCI-level theory, and corresponds to approximately 1 kcal mol^−1^ (≈43 meV per atom). Accordingly, no claim of chemical accuracy in this strict sense is made in the present work.

**Fig. 5:**
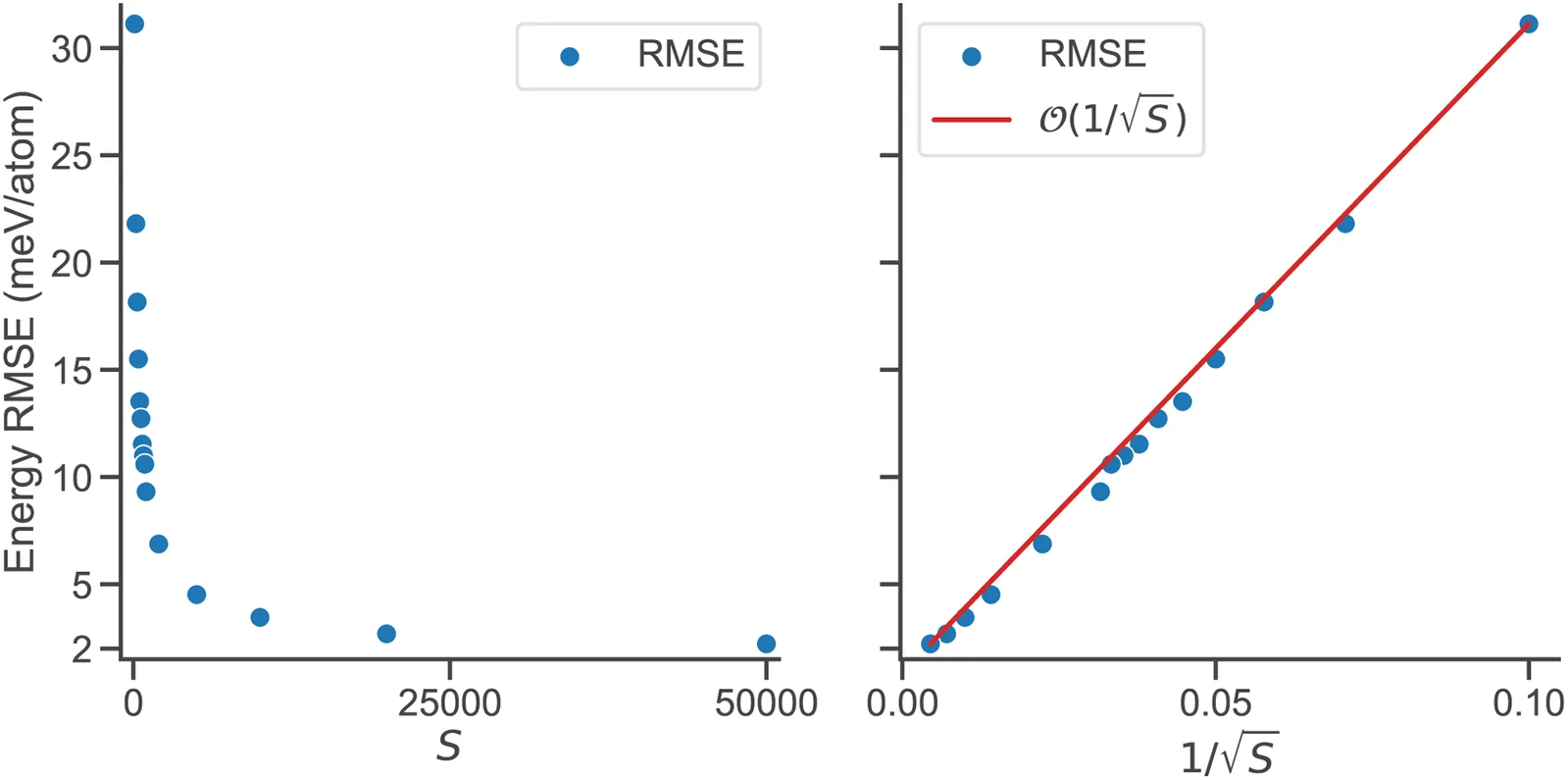
Dependence of HQC-MLP predictions on the number of measurement shots *S*. The root mean square error (RMSE) for the predicted energy (in meV per atom) is measured against the exact, shot-noise-free expectation values of the same trained model, and was estimated using the testing dataset. It therefore isolates the sampling contribution and excludes the model error relative to PBE-DFT. The RMSE decreases with 
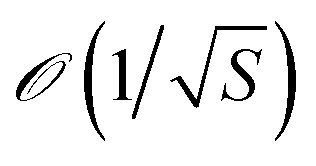
.

A critical consequence of finite shots is the difficulty of atomic force predictions. While gradient estimation methods such as the parameter-shift rule are compatible with shot-based quantum measurements, atomic positions enter the circuit only through the encoding angles, which are functions of the invariant node features produced by the classical network. Forces therefore factorize *via* the chain rule into a classical Jacobian, obtained by backpropagation at no sampling cost, and quantum gradients with respect to the eight encoding angles, which require the parameter-shift rule. This adds 16 shifted circuit executions per readout circuit, resulting in 5120 (16 × 320) circuits per configuration, or ≈2.56 × 10^8^ total shots for a single configuration force evaluation at the budget of 5 × 10^4^ shots per circuit. Repeating this procedure at every MD step would render direct force evaluation prohibitively expensive on current NISQ hardware.^88^ This limitation motivates the use of Monte Carlo simulations, which require only energy evaluations and therefore avoid the expensive computation of atomic forces. We performed canonical MC simulations of liquid silicon at 2000 K using the Metropolis–Hastings algorithm^82^ (Fig. 6). The calculated RDF converges systematically toward the exact (shot-noise-free) result as *S* increases.

**Fig. 6:**
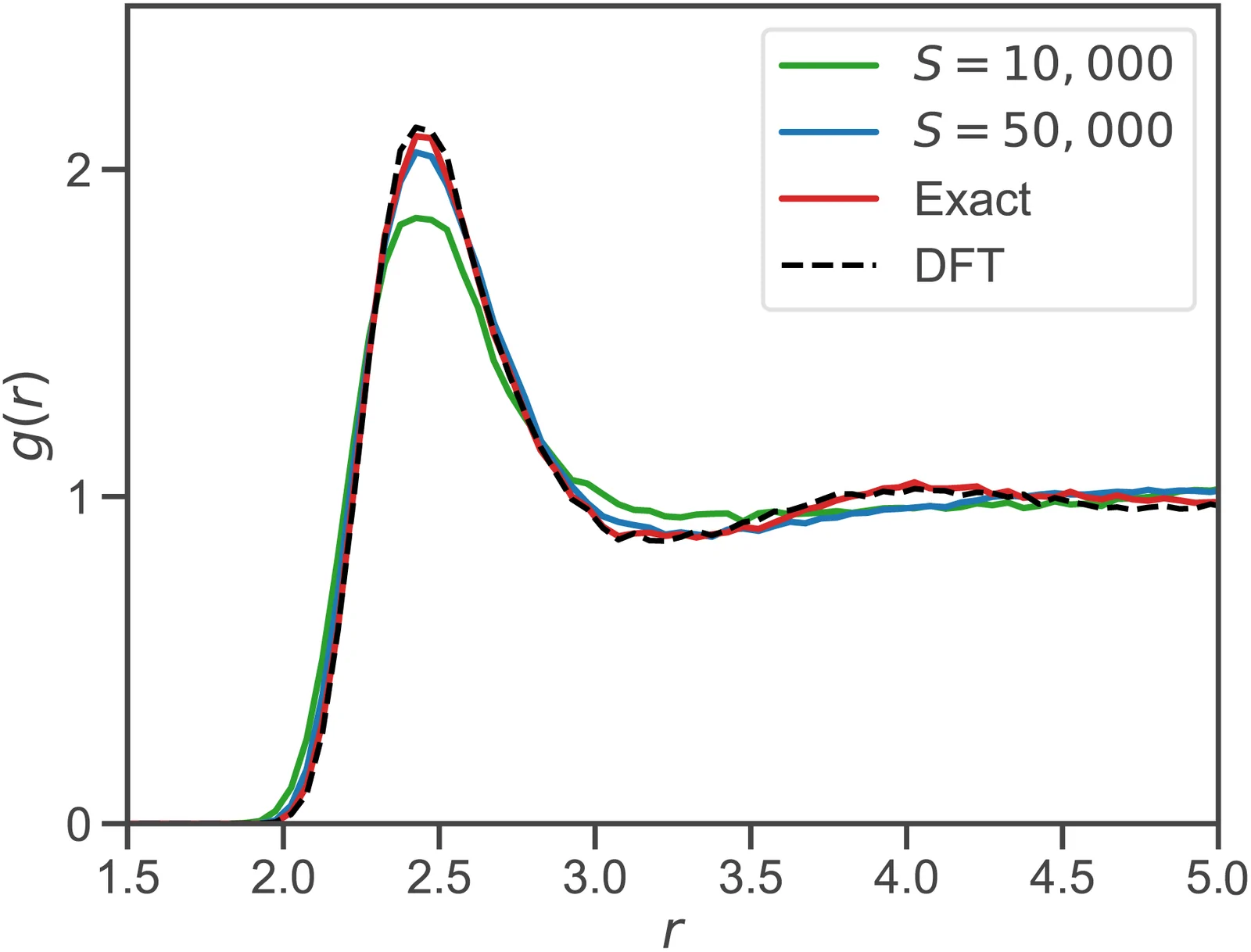
Dependence of the radial distribution function (RDF) of liquid silicon at *T* = 2000 K on the number of measurement shots *S*. The RDF was computed from canonical Monte Carlo (MC) simulations using the BasicEntanglerCX model. As *S* increases, the finite-shot RDFs converge systematically toward the exact (shot-noise-free) RDF. The DFT reference (black dashed curve) was obtained from the independent BOMD trajectory described in Section 4.3.

The MC-based HQC-MLP protocol is in principle within the shot budget of near-term noisy intermediate-scale quantum (NISQ) devices such as IonQ Aria. We note that this assessment is based on an ideal state-vector simulator with finite-shot noise only; a full deployment on real hardware would additionally require characterization of gate errors, decoherence, and readout errors, which are beyond the scope of the present study. The transition from MD to MC simulations entails important physical limitations. Most critically, canonical MC simulations do not yield dynamical information: transport coefficients (*e.g.*, diffusion constants, viscosity), velocity autocorrelation functions, and time-resolved structural properties are inaccessible. Additionally, MC sampling efficiency is sensitive to the choice of trial-move amplitude, which must be tuned separately for each system and state point, whereas MD forces provide a natural guide for trajectory evolution. These limitations mean that, at present, the HQC-MLP protocol with shot-noise-limited hardware is well suited for computing equilibrium structural properties, as demonstrated here for the RDF, but not for studying dynamical phenomena. Resolving this constraint will require either hardware advances that enable practical force evaluation or the development of shot-noise-tolerant force estimators, such as adjoint differentiation or Hellmann–Feynman theorem-based approaches.^88^ While this exceeds current NISQ device throughput, mid-circuit measurement and reset capabilities and parallel qubit operation across multiple circuit copies would make it feasible.

### 4.5 Scalability and scope

A critical question for practical utility is scalability to larger systems and more expressive circuits. In the current architecture, 8 qubits encode the invariant node features. State-of-the-art MLPs commonly use larger feature widths (*F* = 128–256), and encoding these would require correspondingly more qubits and, more critically, deeper circuits. The closed nearest-neighbour entangler employed here applies *n* two-qubit gates per layer, so the gate count per layer grows only linearly; correlations, however, propagate one site per layer, so distributing information across the full register requires a depth of order *n*/2. Correlating *n* qubits therefore requires 
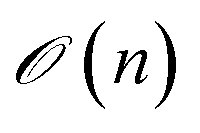
 circuit depth and 
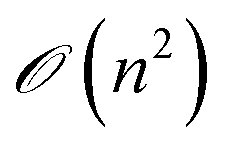
 entangling gates. The three-layer circuits used here are already below this threshold at *n* = 8, so widening the register at fixed depth would leave most encoded features mutually uncorrelated; maintaining the same degree of feature mixing at *F* = 128–256 would require a proportionate increase in depth, with the attendant increase in decoherence and gradient concentration. Both trends increase susceptibility to hardware noise and to the barren plateau problem. We emphasize that barren plateaus are fundamentally a large-*n* phenomenon, and the 8-qubit circuits studied here are too small to probe that regime. Our observation that training gradients did not flatten is therefore consistent with the small system size and should not be read as evidence that the hybrid framework is immune to barren plateaus at scale. Joint optimization of the classical and quantum parameters may partially mitigate this, since AngleEmbedding prepares a product state and so avoids the input entanglement known to induce gradient concentration.^83^ We note, however, that the classical network does not alter the circuit ensemble from which the exponential suppression originates, so it cannot be expected to remove barren plateaus at scale; a systematic study of gradient variance as qubit count and circuit depth increase is required to establish how the approach behaves in the large-*n* qubit limit.

A second, independent scaling axis is the system size *N* (or the number of atoms). Because the VQC acts on per-atom node features, enlarging the simulation cell leaves the circuit width and depth unchanged and increases only the number of circuit executions, which grows linearly as 5*N* per configuration. A 1000-atom cell would therefore require ∼5 × 10^3^ executions and ∼2.5 × 10^8^ shots per energy evaluation at the budget of Section 4.4, with no additional qubit or depth requirement. The hardware constraint on system size is thus one of device throughput rather than of register size.

A third axis is chemical diversity. The HQC-MLP applies to multicomponent systems with the same scheme as the CMLP. Both share the classical network, our Bayesian equivariant potential BAM-RACE, which has been applied to small organic molecules composed of C, H, N, O, and F and to boron nitride in its hexagonal, cubic, and liquid phases.^89^ Atomic species enter through a learned embedding of the node features, and because the two models differ only in the readout, the representation carries over without modification. The one difference is the readout width: the CMLP projects the 64 invariant channels onto 32 inputs of its SiLU-gated readout, whereas the HQC-MLP projects them onto eight circuit inputs, a width set by the qubit count rather than by the chemistry. For elemental silicon the eight-wide readout matches the classical baseline, so the compression is not limiting there. Whether eight projections suffice for chemically heterogeneous environments is an open question, but because the compression is a linear map on the classical side, it can be tested at classical cost by narrowing the CMLP readout on a multicomponent dataset before any circuit is involved; if a wider readout is needed, the qubit and depth costs are those discussed above.

The limitations of the five Ansätze studied here also point toward concrete architectural alternatives. The current circuits inject classical data only once at the start (AngleEmbedding) and use only closed nearest-neighbor entanglement rings with a fixed gate set (RX rotations). Recent theoretical work has demonstrated that data re-uploading, which injects classical features at multiple points within the circuit, allows quantum circuits to act as universal function approximators and enriches the accessible frequency spectrum without requiring additional qubits.^90,91^ A candidate Ansatz motivated by these principles would incorporate all-to-all CZ entanglement with mid-circuit data re-uploading, full Bloch sphere coverage *via*RY + RZ gates, and parameterized *θ*-CZ entanglers. Such a design would remain deployable on IonQ Aria (25 qubits). We present it as a design proposal only, since it has not been trained or tested in the present work, and we leave its implementation and benchmarking to future work.

The present HQC-MLP learns a single ground-state potential-energy surface, and a quantum readout does not by itself provide excited-state energies or nonadiabatic couplings. For the classical model this extension has been demonstrated: BAM-RACE trained on SI-SA-REKS excited-state calculations, with a phaseless loss on the square of the off-diagonal Hamiltonian element, reproduced *ab initio* excited-state molecular dynamics of the penta-2,4-dieniminium cation.^92^ The same scheme carries over to the HQC-MLP by replacing the SiLU-gated readout with the circuit, as done here for the ground state: the linear layer after the circuit can be widened to yield several state-specific outputs from the same circuit evaluations, and the learned Hamiltonian elements are rotationally invariant scalars, which the circuit can provide, with adiabatic energies and coupling vectors following by diagonalization and differentiation. Excited-state accuracy and suitability for nonadiabatic dynamics remain to be demonstrated for the HQC-MLP, and on hardware the gradients of additional states and couplings would add to the measurement cost of Section 4.4.

## 5 Conclusion

In this work, we developed and systematically evaluated a series of HQC-MLPs for simulating high-temperature (2000 K) liquid silicon, representing, to the best of our knowledge, the first application of HQC-MLP methods to a condensed matter system with 64 atoms in a periodic cell. Our findings demonstrate that these models, which couple an *E*(3)-equivariant MPNN with a VQC readout, can accurately reproduce the DFT potential energy surface, validated through stable canonical MD simulations that closely replicate the radial distribution function and, to within 1% of its weight, the bond-angle distribution.

The investigation reveals a nuanced trade-off between training speed and generalization. All HQC-MLP models with entanglement converge faster in epoch count than the classical baseline, and entanglement – rather than VQC structure alone – is responsible for this speedup (demonstrated by the Basic circuit control). However, faster convergence is accompanied by earlier overfitting. The choice of entangling gate is the paramount architectural variable: CZ gates show a consistent trend toward lower validation loss than CX gates across all circuit designs tested. Two mechanisms are consistent with this trend. First, the CZ gate's conditional phase-flip preserves amplitude information while introducing phase correlations. Second, because the final CZ layer commutes with the Pauli-Z measurement operators, it becomes operationally inactive, reducing the circuit's effective entangling depth. Together, these observations support the same design principle, consistent with circuit expressibility theory:^63,64^ the entanglement structure should be matched to the smoothness of the target regression task, and reductions in effective circuit depth arising from gate-measurement commutation should be taken into account when comparing different entangler families for VQC-based regression.

Our analysis of finite-shot measurements establishes quantitative hardware benchmarks for HQC-MLP. Achieving a sampling-induced RMSE_E_ of approximately 2 meV per atom requires about 5 × 10^4^ shots per circuit execution, corresponding to about 1.6 × 10^7^ shots for a single energy evaluation of one atomic configuration. This shot requirement renders direct force calculation prohibitively expensive for MD on current NISQ hardware. In contrast, the MC-based HQC-MLP protocol, which relies solely on energy evaluations, is in principle compatible with near-term quantum devices, provided that realistic hardware noise is appropriately characterized and accounted for. The cutoff radius sensitivity analysis confirms that *r*_cut_ = 4.5 Å is physically well-motivated for liquid silicon, and that HQC-MLP and CMLP exhibit comparable sensitivity to environmental truncation, with differences within seed-variability noise.

Several directions merit future investigation. First, comparing the circuit readouts with a classical readout of matched input width and trainable cross-feature mixing, for example, a small multilayer readout acting on the same eight projected inputs, would sharpen the comparison of the two forms of feature coupling. Extending the gate-type comparison to strongly correlated materials, such as transition metal oxides, Mott insulators, or systems near a metal–insulator transition, will test whether the CZ trend observed here reflects a general design principle or a feature specific to weakly-correlated covalent systems. Incorporating realistic device-noise models beyond the finite-shot analysis presented here is essential before deployment claims on specific hardware can be made. Exploration of more expressive VQC architectures with data re-uploading and parameterized entangling gates is a natural next step. By providing a detailed, quantitative characterization of VQC integration with equivariant MLPs, including transparent null results on accuracy improvement and cutoff robustness, this work establishes a reproducible empirical baseline for the computational chemistry community.

## Author contributions

Soohaeng Yoo Willow: conceptualization, methodology, software, validation, formal analysis, data curation, writing – original draft, visualization, funding acquisition. D. ChangMo Yang: conceptualization, software, writing – review & editing, validation, formal analysis. Kwang S. Kim: formal analysis, validation, writing – review & editing. Chang Woo Myung: conceptualization, methodology, writing – review & editing, visualization, formal analysis, supervision, project administration, funding acquisition.

## Conflicts of interest

There are no conflicts to declare.

## Supplementary Material

RA-016-D6RA07180E-s001

## Data Availability

All data and code supporting this study are openly available on GitHub at https://github.com/myung-group/BAM-QML under the MIT License. The repository contains the CP2K input files used to generate the BOMD reference data, the training and validation trajectories, the model configuration file for each architecture, the training results, and the scripts used to produce the figures in this work. Supplementary information (SI): provides details of the simulation architecture, as well as additional data and analyses supporting the findings discussed in the main text. See DOI: https://doi.org/10.1039/d6ra07180e.
