# Supplementary material for: Hybrid quantum-classical machine learning potential with variational quantum circuits
Source: RSC Adv. 2026 Oct 26. Online ahead of print. doi: 10.1039/d6ra07180e (PMC13699518; doi:10.1039/d6ra07180e)
Supplement: Supplementary file 1 [file RA-OLF-D6RA07180E-s001.pdf]

Supplementary Information (SI) for RSC Advances.
This journal is © The Royal Society of Chemistry

Supplementary Information: Hybrid Quantum–Classical Machine Learning Potential with Variational Quantum Circuits

Soohaeng Yoo Willow*[1], D. ChangMo Yang[1], Kwang S. Kim*[2], and Chang Woo Myung*[1,3,4]

[1] Department of Energy Science, Sungkyunkwan University, 2066 Seobu-ro, Suwon, Gyeonggi-do 16419, Korea.
[2] Department of Chemistry, Ulsan National Institute of Science and Technology, 50 UNIST-gil, Ulsan 44919, Korea
[3] Department of Energy, Sungkyunkwan University, 2066 Seobu-ro, Suwon, Gyeonggi-do 16419, Korea
[4] Department of Quantum Information Engineering, Sungkyunkwan University, 2066 Seobu-ro, Suwon, Gyeonggi-do 16419, Korea
*Authors to whom any correspondence should be addressed.

*Email: sy7willow@skku.edu, kimks@unist.ac.kr, cwmyung@skku.edu

S1 Rotationally equivariant tensor product

The tensor product $\otimes$ in the interatomic continuous-filter convolution f_{Conv} is given by:

$$[e_{ij}]^{l_e}_{m_e} \otimes [h_j]^{l_h}_{m_h} = \sum_{m_e, m_h} C^{l_o, m_o}_{l_h, m_h, l_e, m_e} [e_{ij}]^{l_e}_{m_e} [h_j]^{l_h}_{m_h} \rightarrow [O_{ij}]^{l_o}_{m_o}, \tag{S1}$$

where "rotational order" $l = 0, 1, 2, \ldots$ is a non-negative integer. The representation index m takes values $m \in [-l, l]$. C indicates the Clebsch-Gordan coefficients.

S2 Simple Hybrid Quantum-Classical Architecture

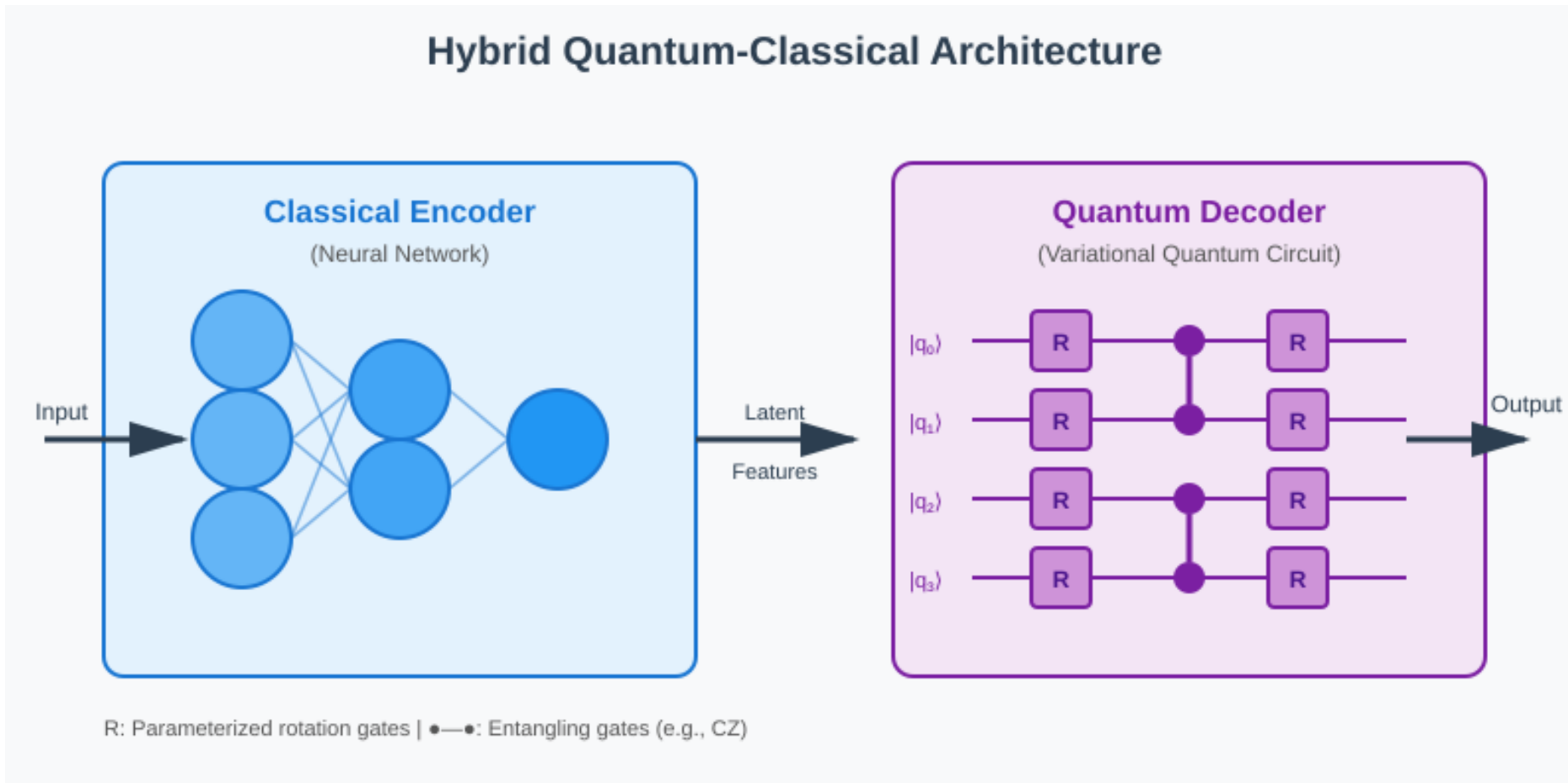

Figure S1: A simple model of the hybrid quantum-classical machine learning potential (HQC-MLP). The classical neural network, based on the NequIP architecture, functions as the encoder. The quantum neural network works as the decoder and predicts the total energy E. The forces acting on the atoms are computed from the energy using $\mathbf{f}_{i\alpha} = -\partial E/\partial \mathbf{r}_{i\alpha}$, where i labels the atoms and α denotes the spatial direction.

In the simple architecture of the hybrid quantum-classical machine learning potential shown in Figure S1, the classical neural network acts as an encoder, while the quantum neural network serves as a decoder that outputs the energy of the system. During training, the model could reproduce the reference energies and forces with good accuracy. The comparison between predicted forces and the DFT forces is shown in Figure S2 (left panel). However, when this trained model was used in molecular dynamics simulations, it failed to capture the correct radial distribution function obtained from DFT, as shown in Figure S2 (right panel). In particular, the HQC-MLP model exhibits an unphysical peak at $r < 2$ Å. This behavior suggests that the short-range repulsive interactions, originally learned by the classical neural network through the learnable radial functions $R(r_{ij})$ in Eq. (2), were weakened or lost in the quantum decoder, possibly due to gradient-flattening effects in the quantum decoder. To resolve this issue, we developed a revised approach and evaluated its performance. The details of this method are presented in the main manuscript.

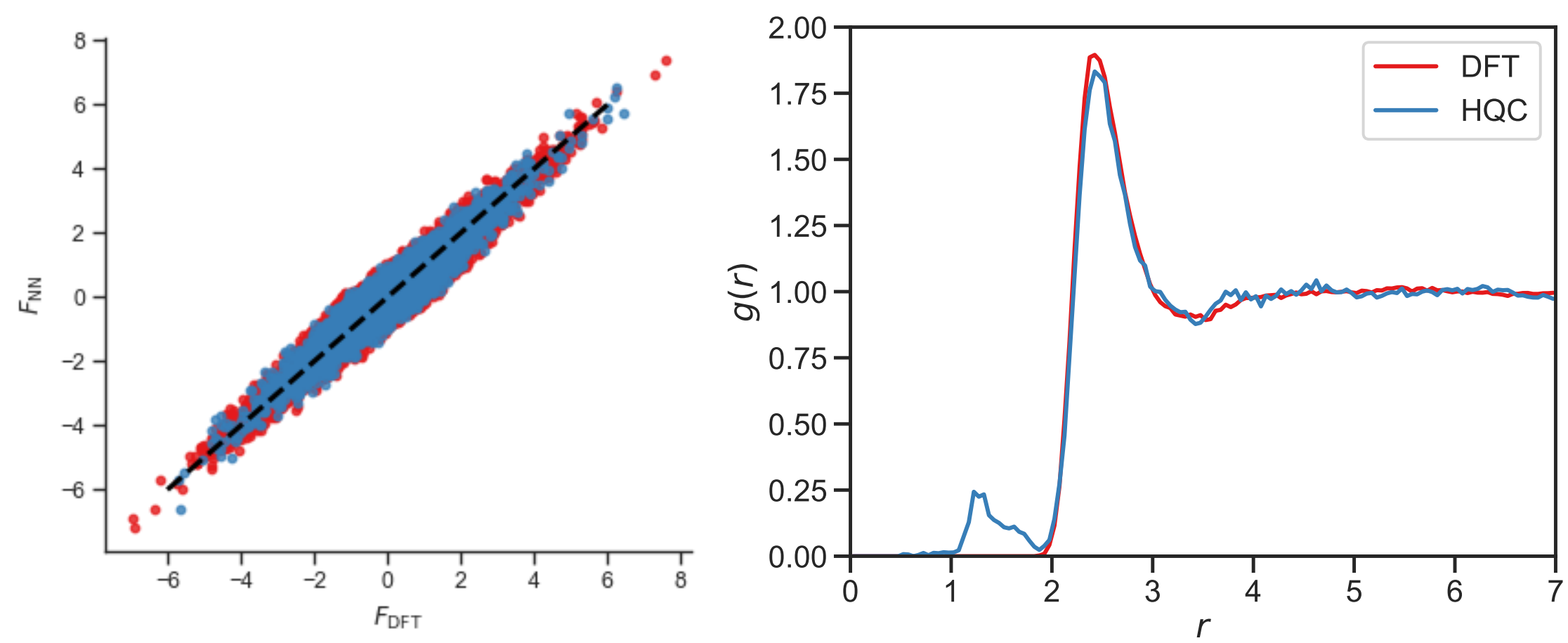

Figure S2: The left panel compares the HQC force prediction $F_{\rm NN}$ with the reference DFT forces $F_{\rm DFT}$. Red and blue dots represent the training and validation datasets, respectively, and indicate that the HQC model is trained successfully. The right panel shows the radial distribution functions obtained from molecular dynamics simulations at $T = 3000$ K using both the HQC model and the DFT reference model. A spurious peak at $r < 2$ Å in the HQC-MLP prediction reveals that the HQC model failed to capture the correct structural features predicted by DFT.

S3 Improved Hybrid Quantum-Classical Architecture

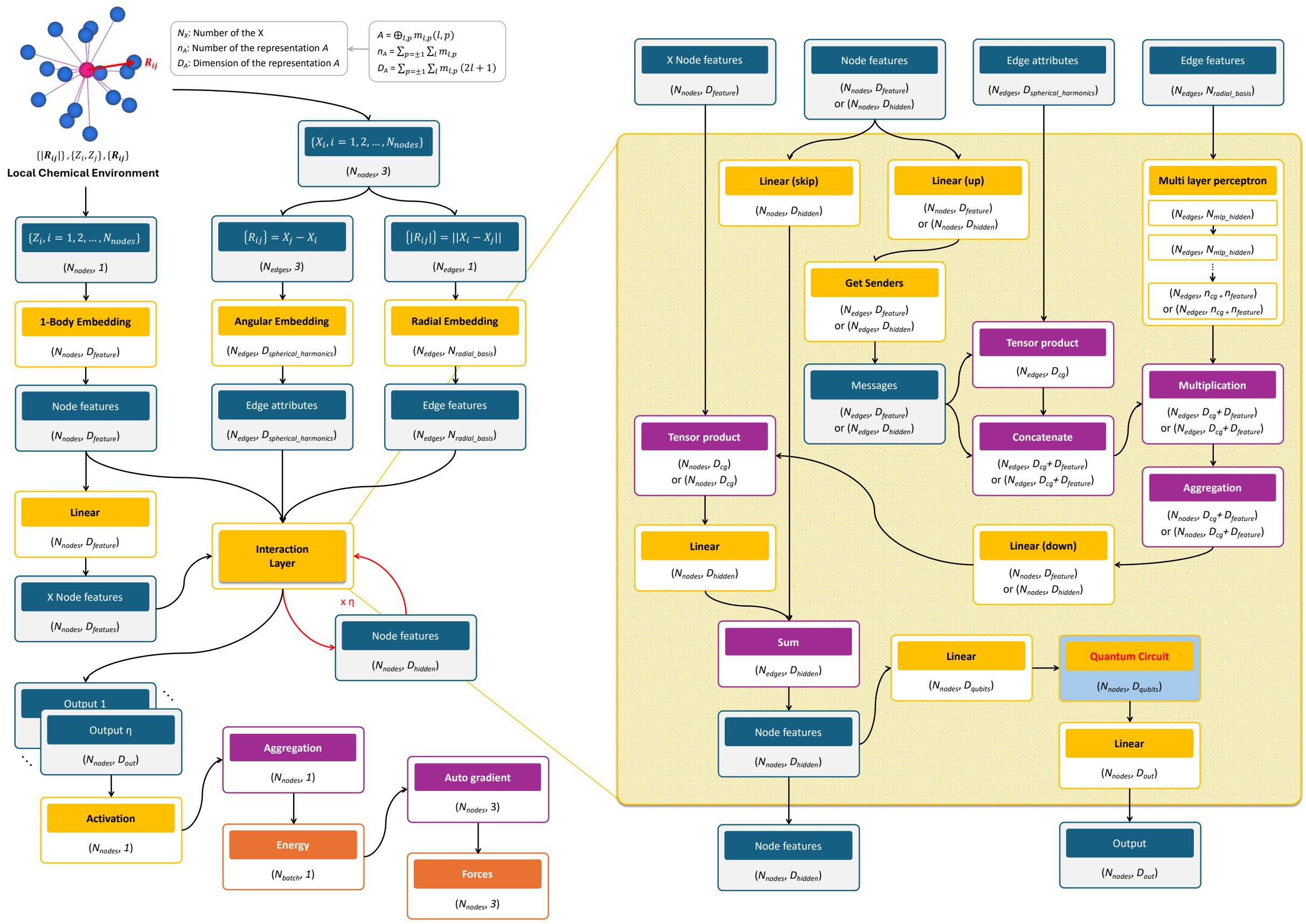

Figure S3: Algorithm flowchart of the improved version of the HQC-MLP model. The blue boxes represent the input or output features, with their corresponding shapes indicated. The yellow and purple boxes denote computational operations, where the annotated shapes indicate the output dimensions of the respective operations. The orange boxes represent the final desired output. The cyan box represents the quantum circuit. X denotes the positional information of atoms in the system (x, y, z coordinates), Z represents the atomic species, and $\boldsymbol{R}$ corresponds to the interatomic distance vectors.

S4 Dependence of Loss profiles on random seeds

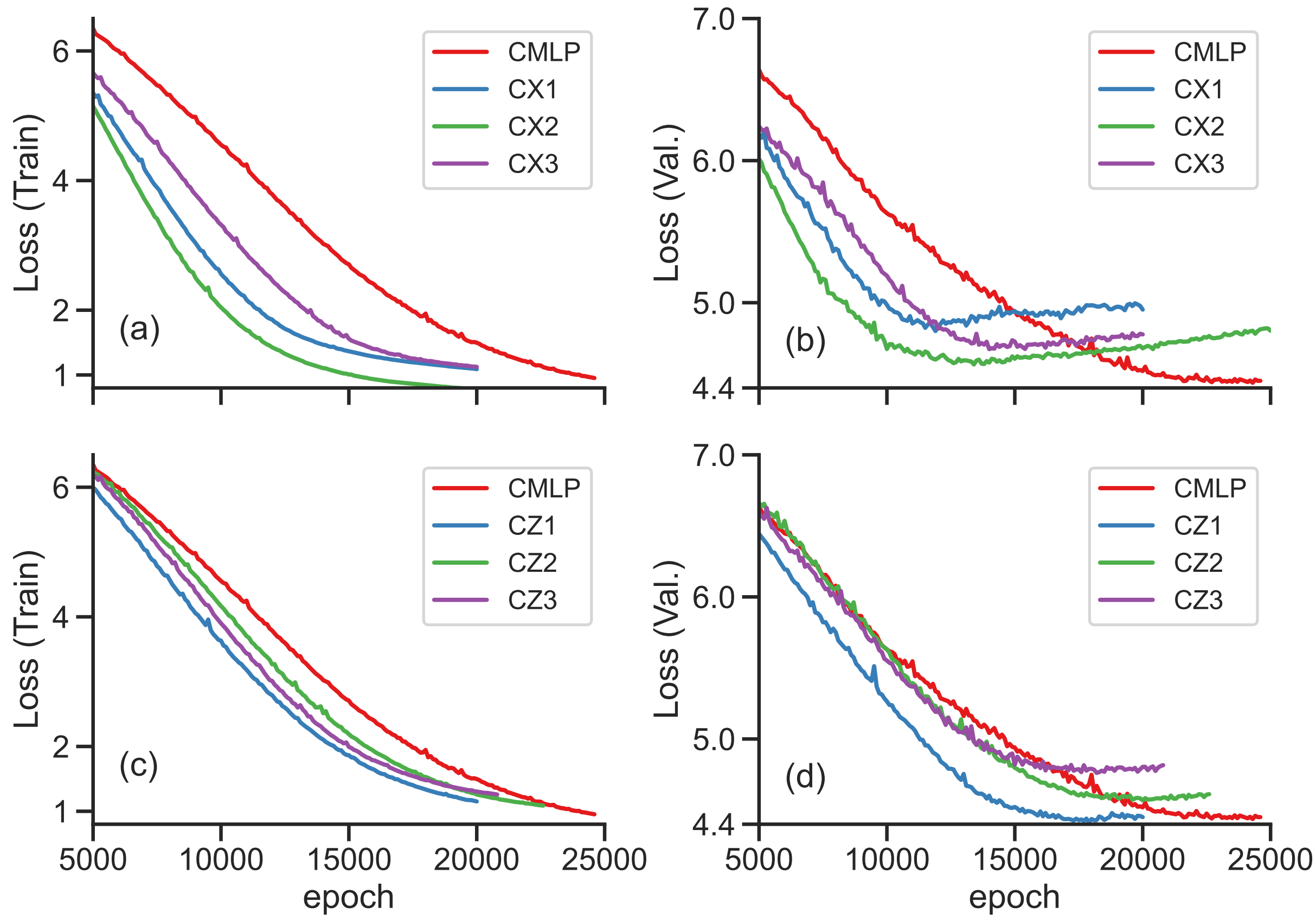

Figure S4: The loss profiles of hybrid quantum-classical machine learning potential (HQC-MLP) models with `BasicEntanglerCX` and `BasicEntanglerCZ` Ansatz modules compared to a classical machine learning potential (CMLP). Panels (a) and (b) show training and validation losses, respectively, for the `BasicEntanglerCX` Ansatz with three different random seeds (CX1, CX2, CX3). Panels (c) and (d) show corresponding results for the `BasicEntanglerCZ` Ansatz with three random seeds (CZ1, CZ2, CZ3). In the validation datasets, the loss values exhibit clear minima, indicating the onset of overfitting as training progresses.

S5 Training convergence via Root mean squared errors on the validation set

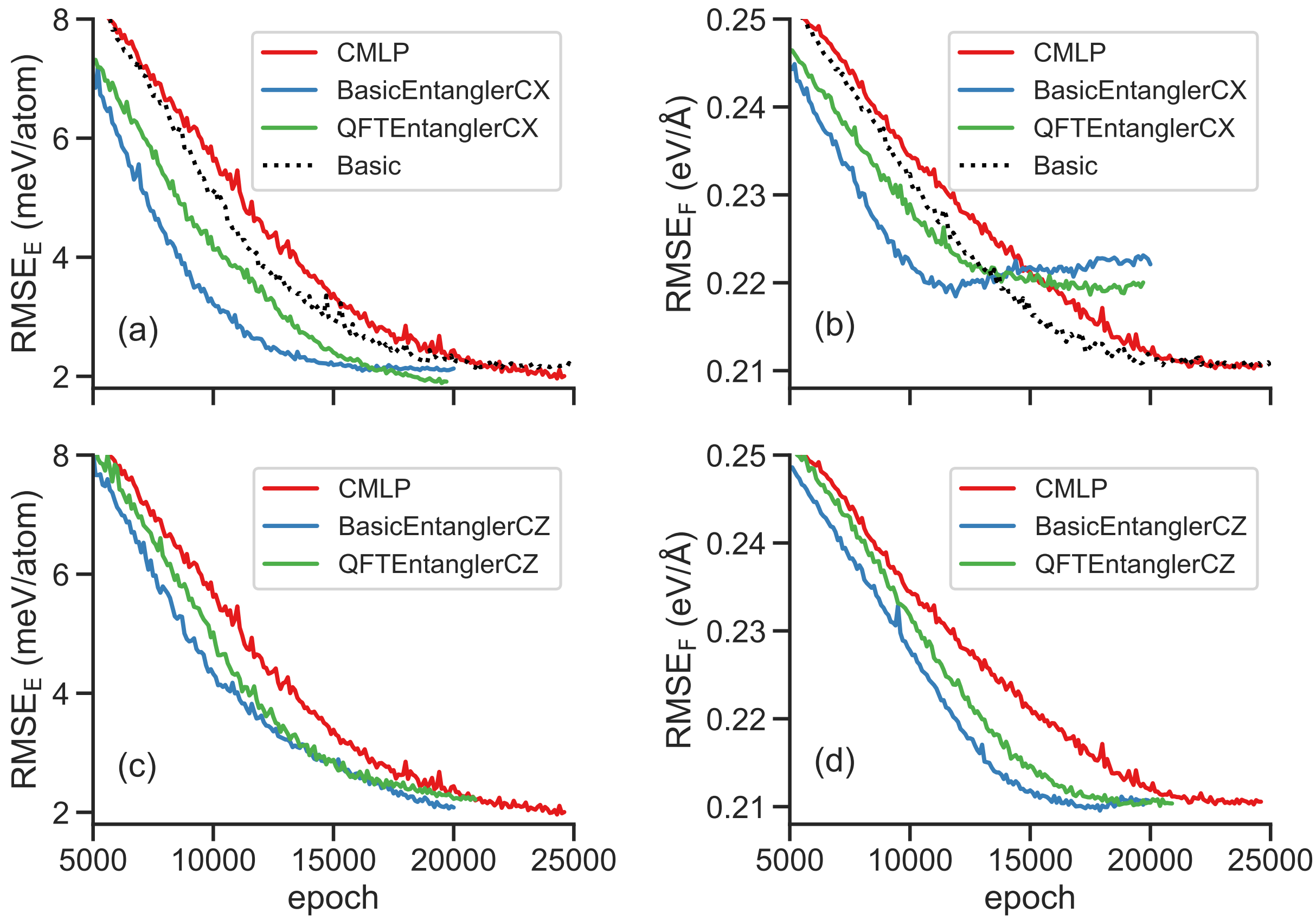

Figure S5: The root mean squared errors (RMSEs) for predicted energies per atom ($\mathrm{RMSE_E}$) and predicted forces ($\mathrm{RMSE_F}$) are evaluated on the validation set and are used here to monitor convergence and to locate the early-stopping checkpoint; the accuracy values reported in the main text are test-set values evaluated at that check point. The HQC-MLP models (blue and green lines) show a faster decrease in both $\mathrm{RMSE_E}$ and $\mathrm{RMSE_F}$ compared to the CMLP model (red line).

S6 Effect of the cutoff radius on the training convergence

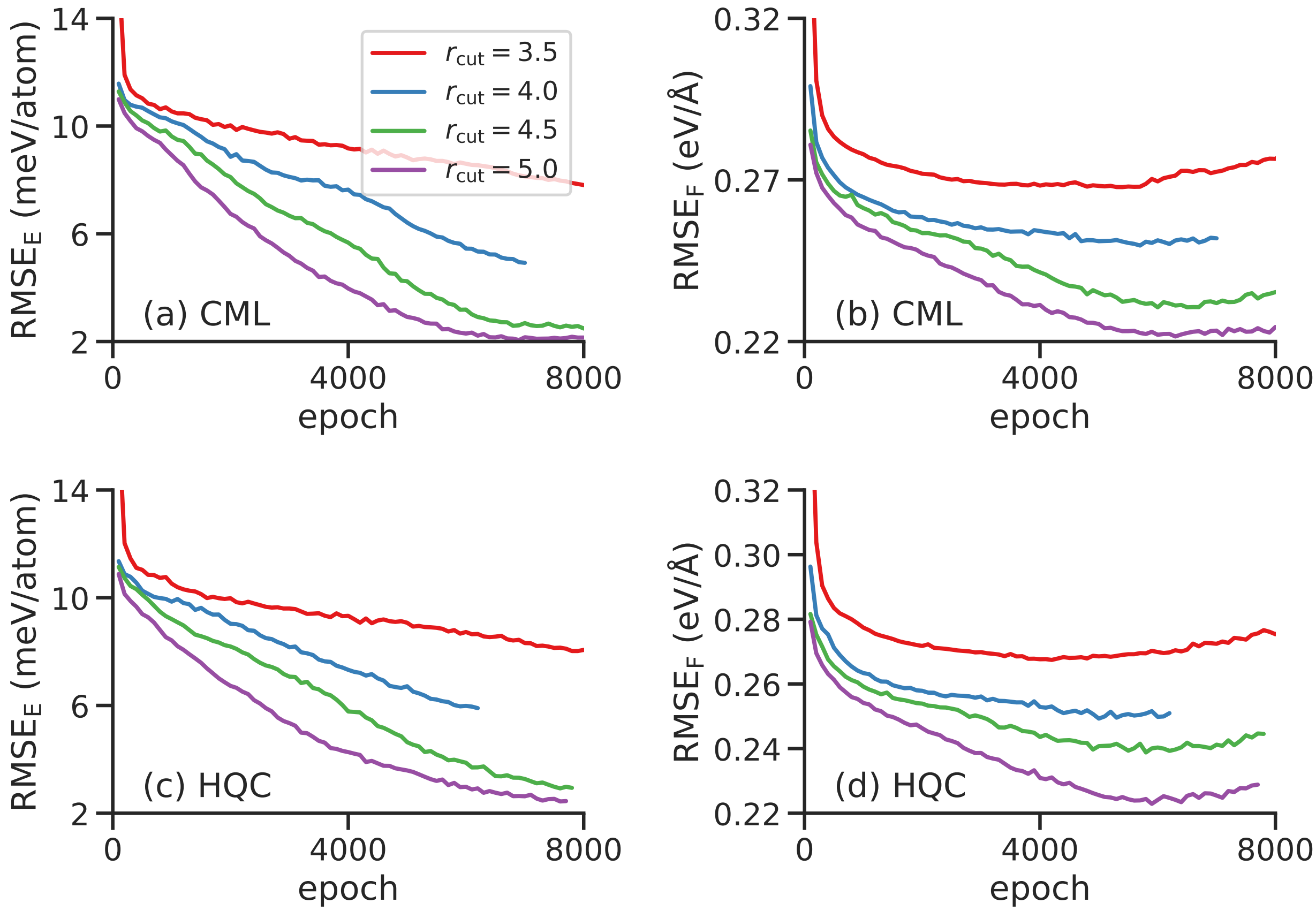

Figure S6: Effect of the cutoff radius (r_{cut}) on the training convergence of the classical (CMLP) and HQC machine-learning potentials. Panels (a) and (b) present the root-mean-square errors of the energy (RMSE$_E$, in meV/atom) and forces (RMSE$_F$, in eV/Å), respectively, as a function of training epoch for the CMLP model, whereas panels (c) and (d) show the corresponding results for the HQC-MLP (**BasicEntanglerCZ**) model. Four cutoff distances are considered in each panel: r_{cut} = 3.5, 4.0, 4.5, and 5.0 Å. For both models, increasing r_{cut} systematically reduces RMSE$_E$ and RMSE$_F$, indicating that incorporating a larger atomic environment improves the accuracy of predicted energies and forces. The calculations shown here were performed using a batch size of 5, whereas other calculations shown in Figures S4 and S5 employed a batch size of 20.

S7 Conservation of the Hamiltonian

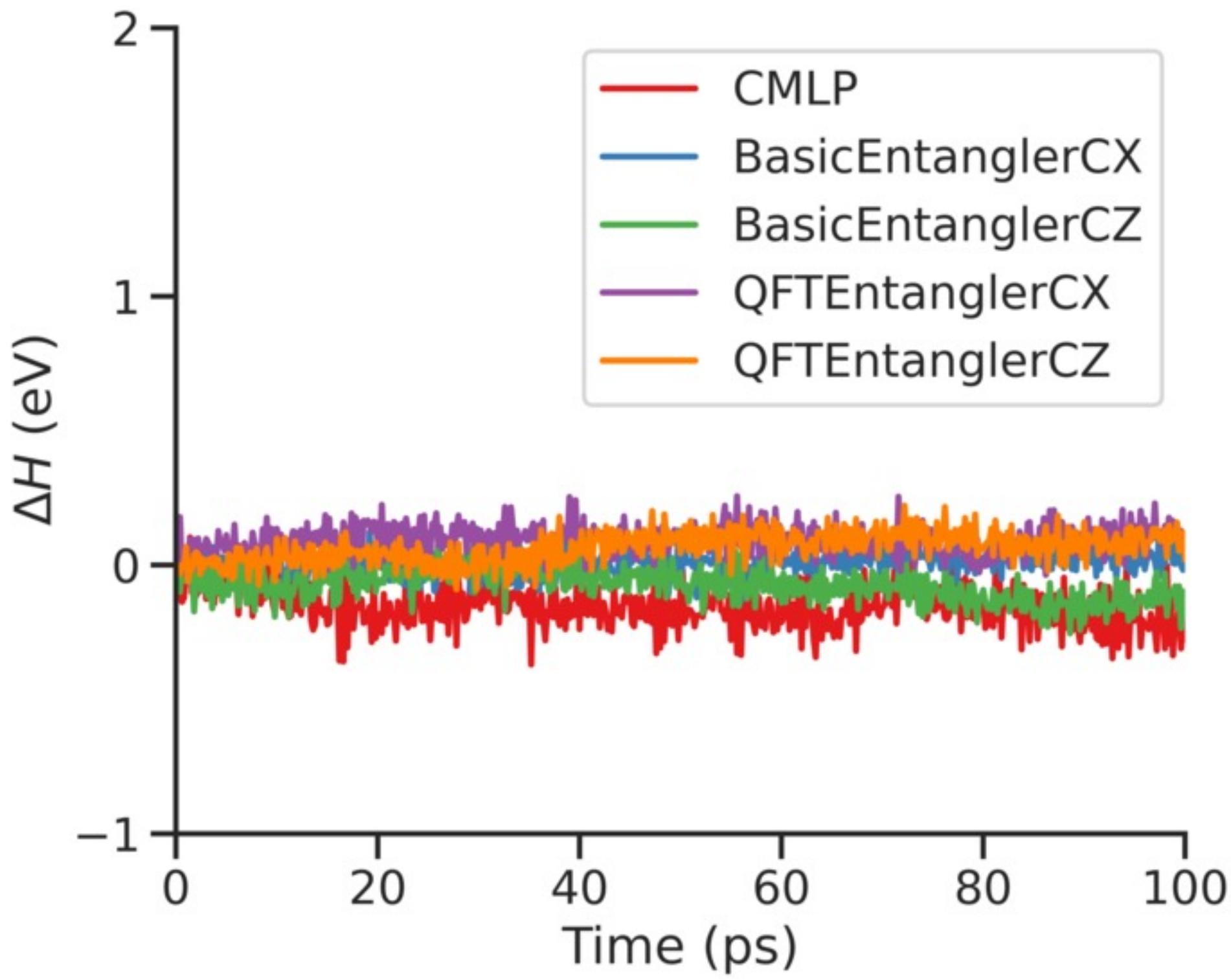

Figure S7: Conservation of the Hamiltonian along canonical MD trajectories at $T = 2000$ K. This temperature was chosen to assess the transferability of the model, which was trained exclusively on configurations at 3000 K. The relative Hamiltonian is defined as $\Delta H = H(t) - H(t = 0)$, where $H(t)$ and $H(0)$ denote the Hamiltonian at time t and its initial value, respectively.

S8 Radial distribution functions

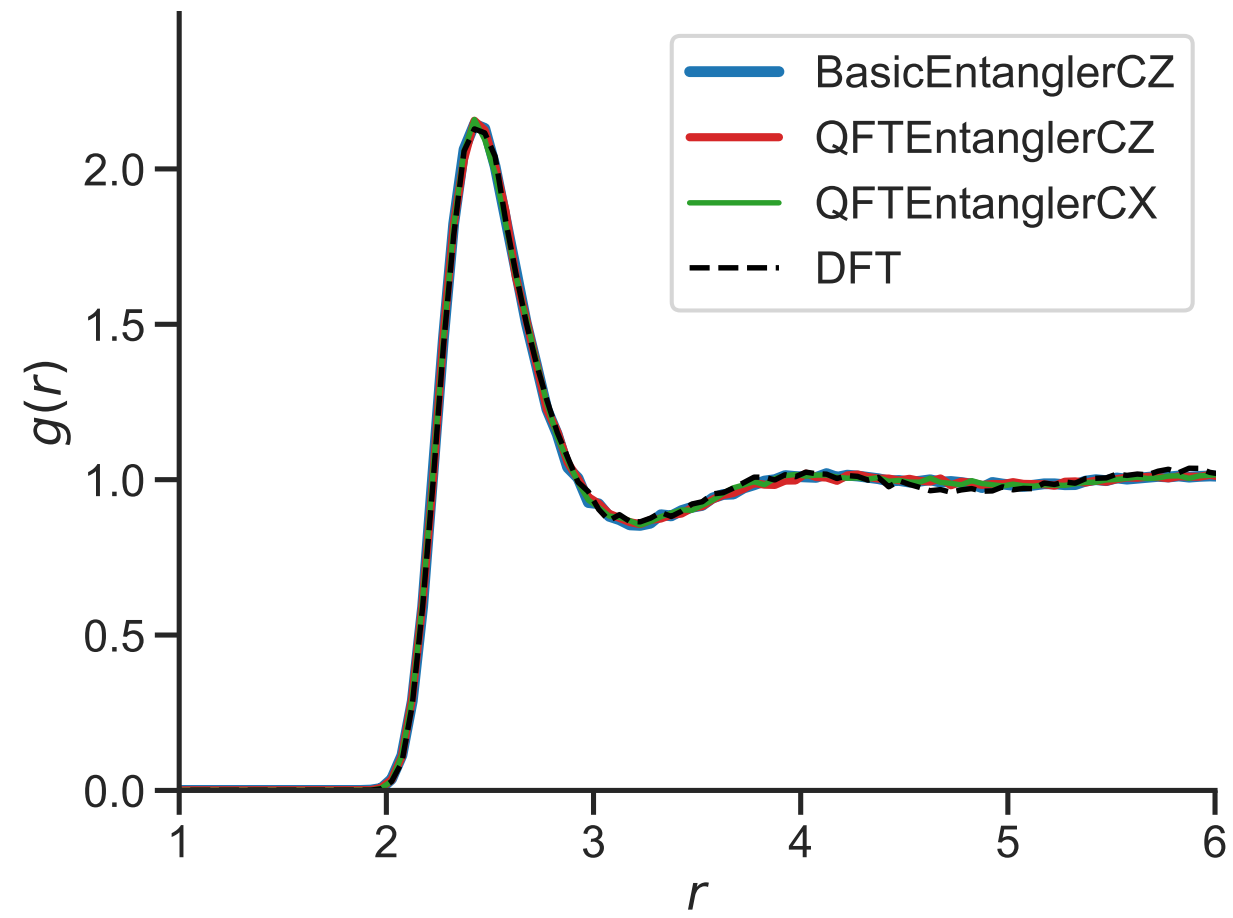

Figure S8: Radial distribution function, $g(r)$, for liquid silicon at $T = 2000$ K as the transferability test. The figure compares the three remaining entangled HQC-MLP variants (`BasicEntanglerCZ`, `QFTEntanglerCZ`, and `QFTEntanglerCX`) with the DFT reference (dashed black line), demonstrating excellent agreement. The result for the fourth variant, `BasicEntanglerCX`, is presented in Figure 4 of the main text.

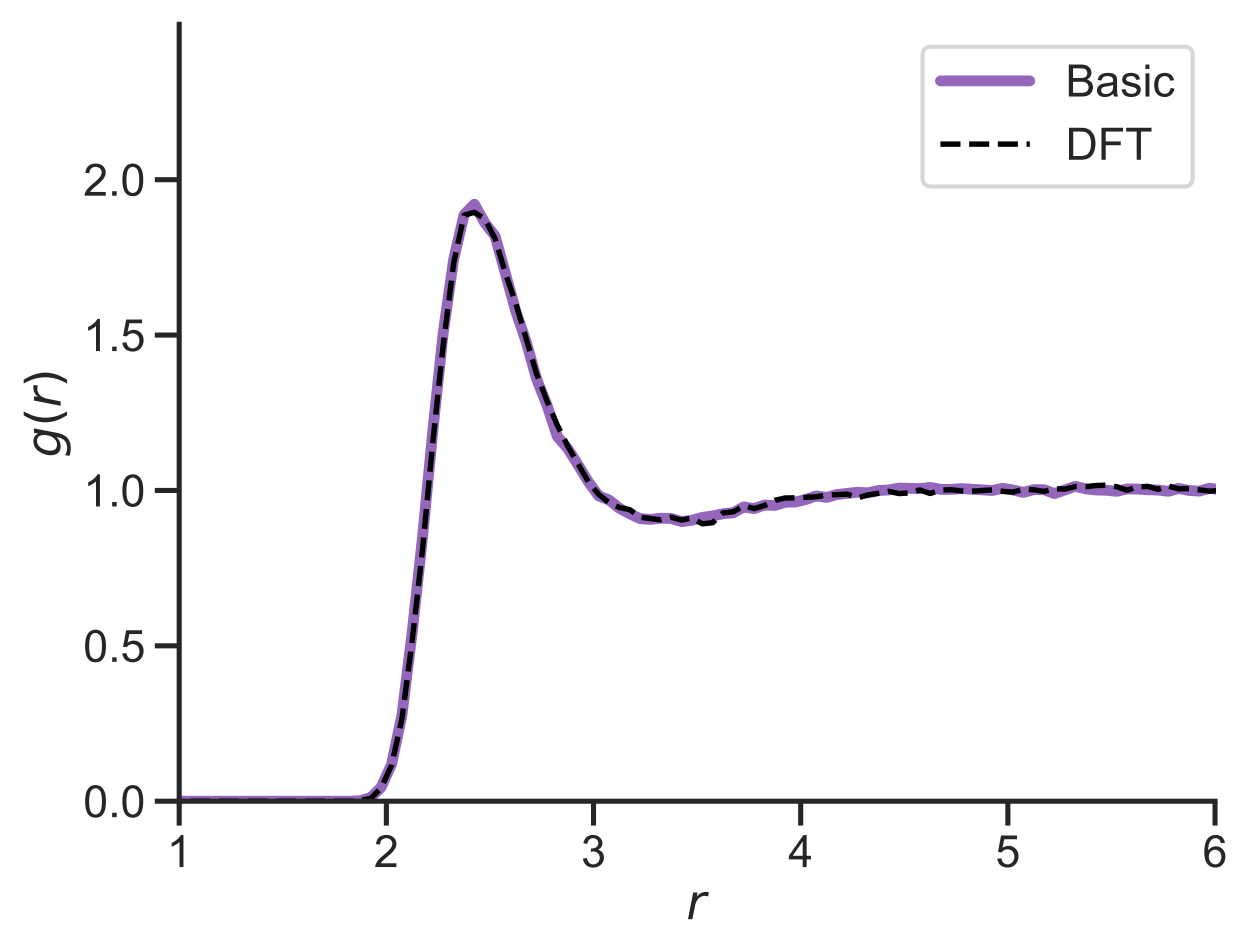

Figure S9: Radial distribution function, $g(r)$, at $T = 3000$ K, i.e. at the temperature of the training data (in-distribution check). The plot compares the results from the HQC-MLP (`Basic`, the non-entangled control circuit) with the DFT reference (dashed black line), demonstrating excellent agreement. The 2000 K transferability comparisons are shown separately in Figure 4 and Figure S8.

S9 Angular distribution functions

Agreement in $g(r)$ does not establish the accuracy of angular correlations. To test these, we compare the Si–Si–Si bond-angle probability density for DFT and the HQC-MLP variants at the same temperature, using identical neighbor definitions and histogram bins. For a central atom i and an unordered pair of distinct neighbors j, k, the angle is

$$\theta_{jik} = \cos^{-1}\left(\frac{\boldsymbol{r}_{ij} \cdot \boldsymbol{r}_{ik}}{|\boldsymbol{r}_{ij}|\,|\boldsymbol{r}_{ik}|}\right). \tag{S2}$$

Here the displacement vectors account for periodic boundaries. Both neighbors must lie within $r_c = 3.24$ Å (3.2375 Å) of atom i. This cutoff is the first minimum of the 2000 K DFT $g(r)$. At 3000 K the minimum is a broad plateau, over which $g(r)$ stays within 0.02 of its minimum value from 3.24 to 3.61 Å; it contains this cutoff, and a single cutoff is used for all models and both temperatures so that every distribution shares the same neighbor definition. The resulting mean DFT coordination numbers are 6.48 at 2000 K and 6.59 at 3000 K. Each unordered neighbor pair is counted once, the angles are binned in 2° intervals, and the distribution is normalized so that $\int_0^{180^\circ} P(\theta)\,d\theta = 1$, with P in degree^{-1} and without division by $\sin\theta$.

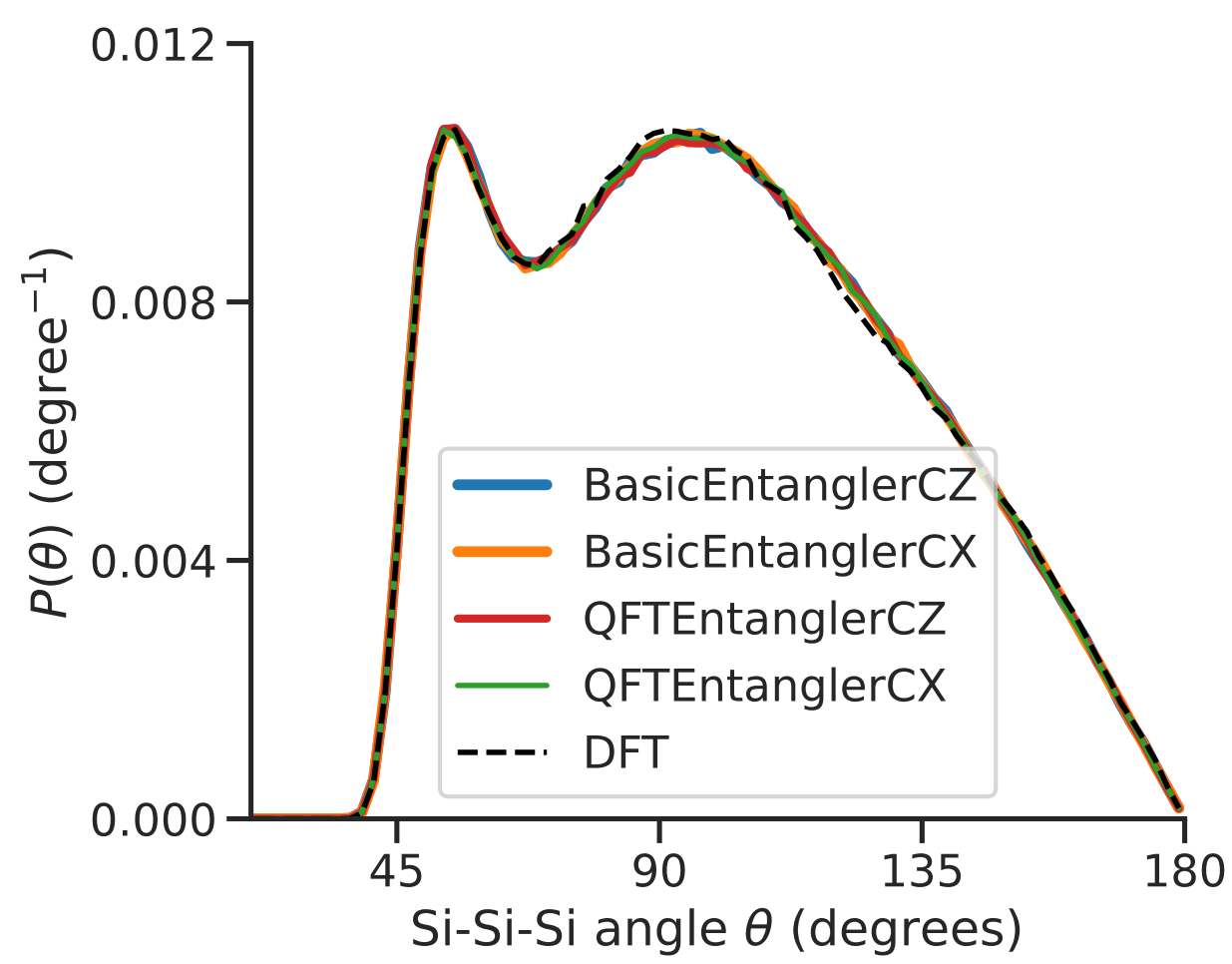

Figure S10: Angular distribution function, $P(\theta)$, for liquid silicon at $T = 2000$ K, the transferability test. The figure compares the four entangled HQC-MLP variants (`BasicEntanglerCZ`, `BasicEntanglerCX`, `QFTEntanglerCZ`, and `QFTEntanglerCX`) with the DFT reference (dashed black line). All four reproduce the two maxima and the mean coordination number. Neighbor cutoff 3.24 Å; bin width 2°.

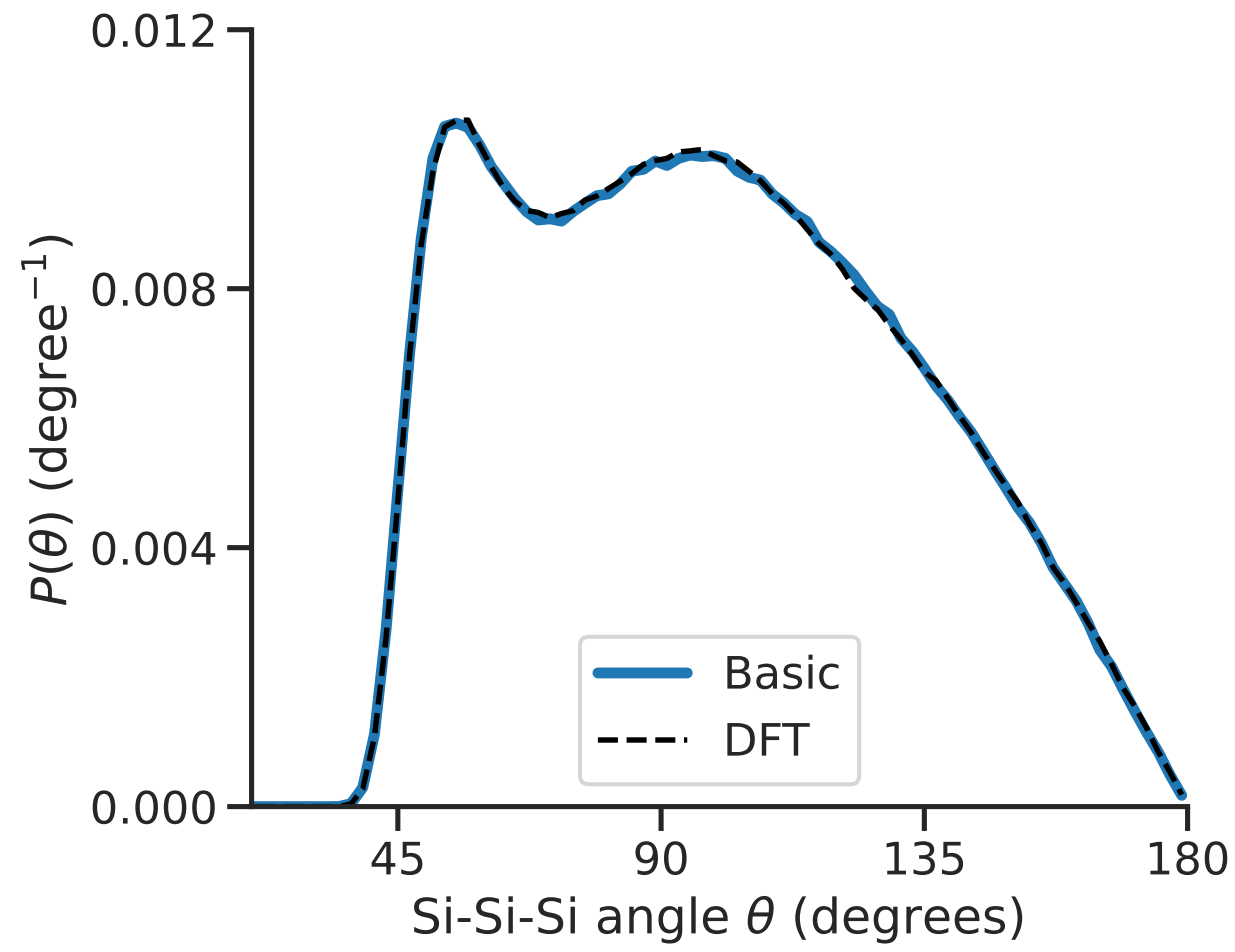

Figure S11: Angular distribution function, $P(\theta)$, for liquid silicon at $T = 3000$ K, i.e. at the temperature of the training data. The plot compares the results from the HQC-MLP (`Basic`, the non-entangled control circuit) with the DFT reference (dashed black line); the two differ by less than the two halves of the DFT trajectory differ from each other. This comparison is an in-distribution check; the transferability test at 2000 K is shown in Figure S10. Neighbor cutoff 3.24 Å; bin width 2°.

S10 Training cost relative to CMLP

Table S1: Training cost of the HQC-MLP variants relative to the CMLP: wall time per epoch, and wall time to reach a common validation force error, $\mathrm{RMSE}_F \leq 0.222$ eV/Å, the lowest value reached by every model. Per-epoch times are from runs executed consecutively on the same NVIDIA GeForce RTX 4090 GPU with identical training settings. The number of epochs to the target is the first epoch at which the median of five consecutive logged validation values (25 epochs) reaches it.

Model	s/epoch	Per-epoch ratio	Hours to val $\mathrm{RMSE}_\mathrm{F} \leq 0.222$ eV/Å	Time ratio
`CMLP`	8.58	1.00	34.8	1.00
`Basic`	10.69	1.25	38.1	1.09
`BasicEntanglerCX`	10.70	1.25	29.1	0.83
`BasicEntanglerCZ`	11.24	1.31	39.9	1.15
`QFTEntanglerCX`	20.11	2.35	68.5	1.97
`QFTEntanglerCZ`	23.24	2.71	80.4	2.31

S11 Parameters in one readout block

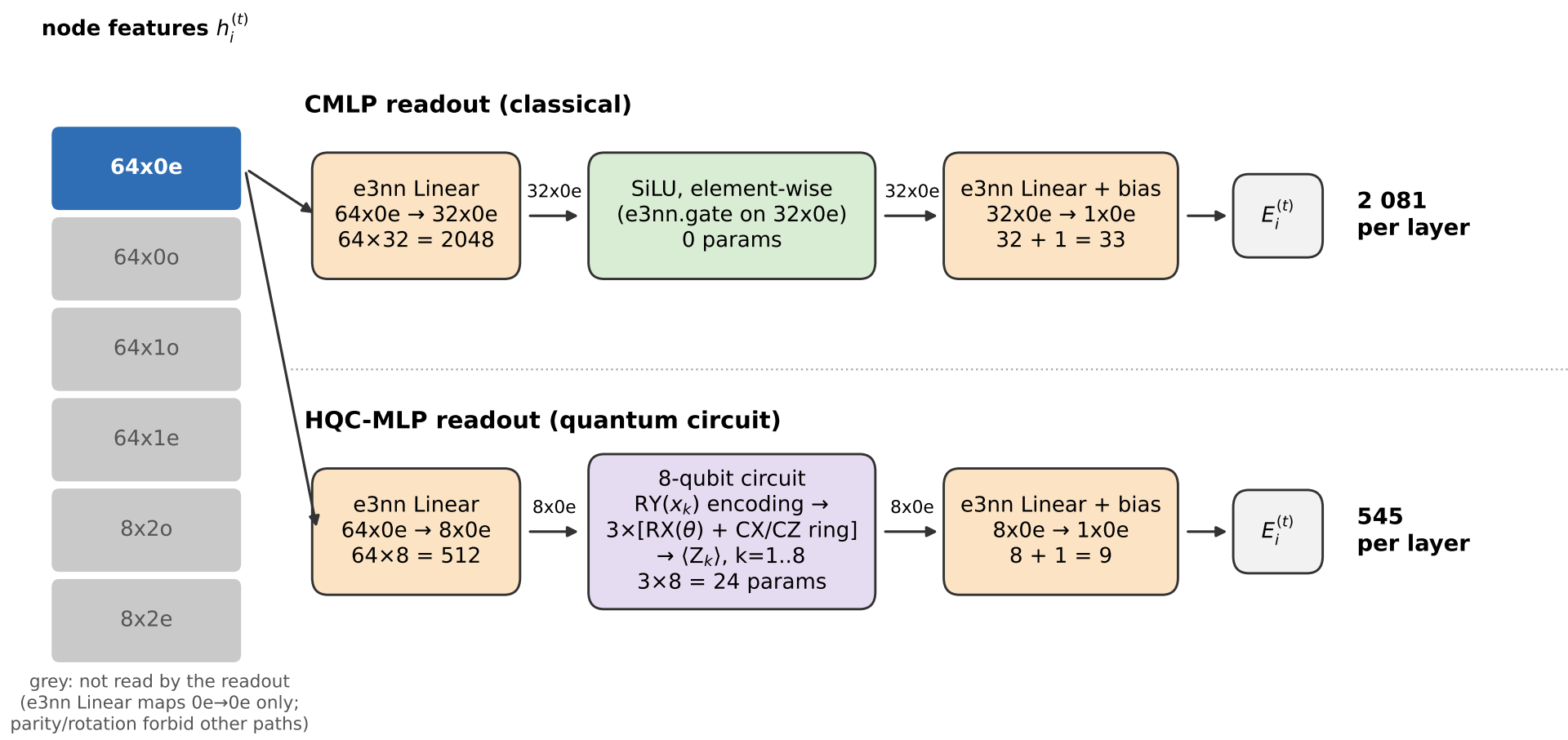

Figure S12: Readout block of one interaction layer in the CMLP (top) and the HQC-MLP (bottom). Both read only the 64 scalar ("0e") channels of the node features; the other irreps have no equivariant linear path to a scalar. The CMLP readout has four times the hidden width (32 against 8 channels) and 3.8 times the trainable parameters (2,081 against 545 per layer; 10,405 against 2,725 over five layers)
